# Functionally Characterizing the Renal Cell Carcinoma Tumor-Immune Microenvironment via Patient-Derived *Ex Vivo* Models

**DOI:** 10.1158/2767-9764.CRC-25-0447

**Published:** 2026-02-26

**Authors:** Neja Sirc, Johannes Smolander, Anita N. Kumari, Jianyin Liu, Moon Hee Lee, Kanerva Lahdensuo, Riikka Järvinen, Sara V. Tornberg, Linh Lin, Hanna K. Laitinen, Jason Theodoropoulos, Jay Klievink, Tuomas Mirtti, Anna S. Kreutzman, Heidi M. Haikala, Petrus Järvinen, Satu Mustjoki, Karita Peltonen

**Affiliations:** 1 Hematology Research Unit Helsinki, University of Helsinki and Helsinki University Hospital Comprehensive Cancer Center, Helsinki, Finland.; 2 Translational Immunology Research Program and Department of Clinical Chemistry and Hematology, University of Helsinki, Helsinki, Finland.; 3 iCAN Digital Precision Cancer Medicine Flagship, Helsinki University Hospital, University of Helsinki, Helsinki, Finland.; 4 HaikaLab Immuno-Oncology Research Group, Translational Immunology Research Program (TRIMM), Research Programs Unit, University of Helsinki, Helsinki, Finland.; 5 Abdominal Center, Urology, Helsinki University Hospital, Helsinki University, Helsinki, Finland.; 6 Department of Pathology, Research Program in Systems Oncology, Helsinki University Hospital, University of Helsinki, Helsinki, Finland.; 7 Finnish Cancer Institute, Helsinki, Finland.

## Abstract

Immune cells in the tumor microenvironment (TME) are attractive therapeutic targets; however, their responses to immunotherapy and targeted therapy remain incompletely understood. We developed a patient-derived *ex vivo* system to profile baseline immune characteristics and model treatment-induced activation at the single-cell level. The model was utilized to study T cell responses to PD-1 blockade and VEGFR inhibition (VEGFRi) in patients with renal cell carcinoma (RCC). The baseline RCC TME was highly infiltrated by T cells, characterized by diverse cytotoxic, memory, exhausted, or regulatory phenotypes. T cells were activated by direct CD3/CD28/CD2 stimulation, upregulating the IFN-γ, TNF, and IL2 signaling pathways. However, activation capacity varied noticeably depending on the baseline phenotype. PD-1 blockade induced modest T cell activation, whereas VEGFRi downregulated several immune markers, including signaling pathways and immune activation-related cytokines. Our study reveals the suppressive features of the RCC TME and the challenge of fully overcoming it with PD-1 blockade and VEGFRi.

**Significance:**

We developed a patient-derived *ex vivo* model to study immune cell therapy responses within the TME of RCC. Immune activation toward PD-1 blockade and VEGFRi was attenuated and depended on the immune cell state.

## Introduction

Renal cell carcinoma (RCC) is the most common type of kidney cancer with a rapidly increasing incidence in developing countries (1). Approximately 70% of cases present with clear cell histology (ccRCC), a subtype recognized for its high immunogenicity (2). This classification was originally established based on the clinical benefits observed from IL2 and IFN-α treatments (3, 4), which suggested a key role of immune system activation in achieving positive treatment responses. Recent clinical trials demonstrated the therapeutic efficacy of immune checkpoint inhibitors in ccRCC and other RCC subtypes (5, 6). Many of these trials have incorporated VEGF inhibitors, a standard-of-care treatment for RCC. The rationale for combining VEGF inhibition with checkpoint blockade is supported by evidence that VEGF not only promotes angiogenesis but also contributes to immunosuppression by altering immune cell recruitment and function within the tumor microenvironment (TME; refs. 7, 8).

RCC is characterized by high immune cell infiltration (9, 10) that is paradoxically associated with worse outcomes (11). This paradox may reflect impaired adaptive immune cell function driven by immunosuppressive cells within the TME. Single-cell analyses have advanced our understanding of immune cell heterogeneity in the RCC TME (12, 13), mechanisms of immune cell dysfunction (14, 15), and identified factors associated with checkpoint blockade therapy (16, 17). However, a comprehensive understanding of the immunosuppressive mechanisms within the RCC TME remains elusive. It is especially relevant to understand whether suppressed immune cells can be effectively reactivated to restore antitumor activity. Furthermore, the immunologic mechanisms underlying the effects of checkpoint blockade therapy, particularly in combination with VEGF inhibition, remain poorly understood.

**Figure 1.**
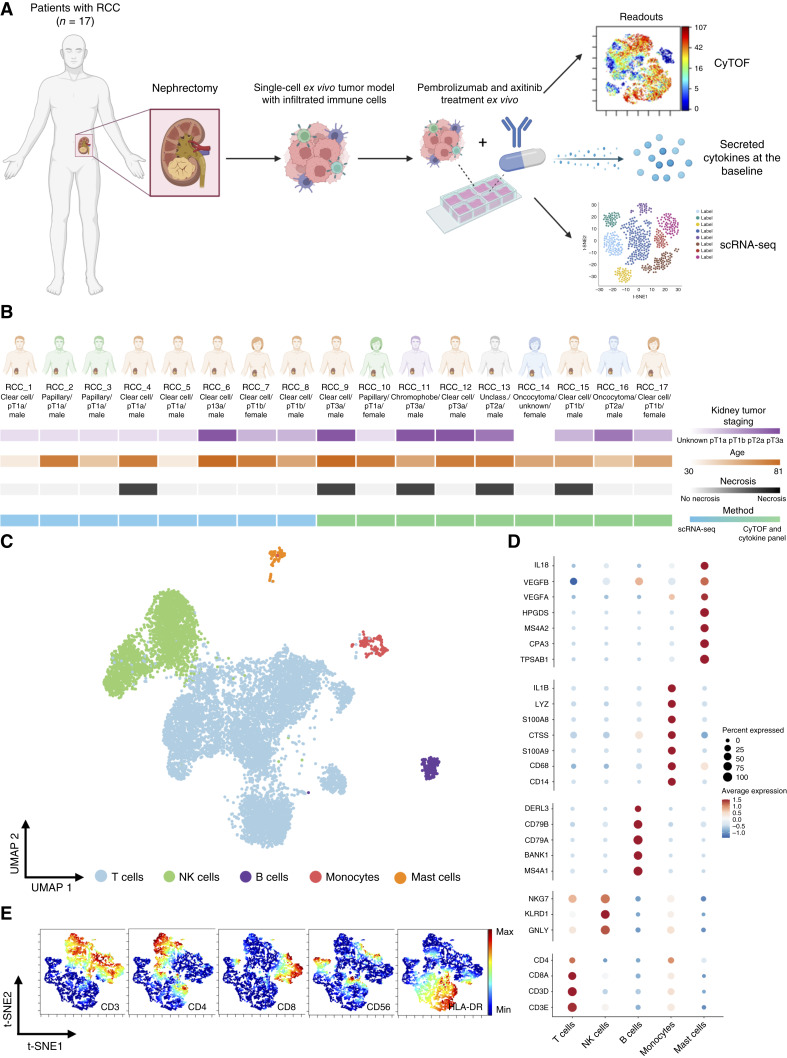
Overview of the study and characterization of CD45^+^ cells. **A,** Study overview for each of the 17 patients. Readout for nine patients was performed applying CyTOF and analysis of secreted cytokines, whereas readout for eight patients was performed with scRNA-seq. **B,** Clinical description of the patient cohort, showing patient ID numbers, diagnosis, stage, sex, age, necrosis level, and the methods applied in the experiments. **C,** UMAP of 10,003 untreated control CD45^+^ cells captured across eight patient samples (RCC_1–RCC_8), representing the baseline, colored by broad cell types. **D,** Marker genes used to define broad cell types (T cells, NK cells, B cells, monocytes, and mast cells). **E,** t-distributed stochastic neighbor embedding (t-SNE) maps displaying 8,172 untreated control CD45^+^ cells from patients RCC_9–RCC_17, representing the baseline, analyzed by CyTOF. Cells colored by expression of indicated markers on the t-SNE map.

To address this gap, we developed a 3D *ex vivo* culture platform using patient-derived RCC tissue (Fig. 1A) to study the immunologic responses to therapy within the TME. Tumor tissue was dissociated and embedded in an extracellular matrix (ECM) to preserve tumor-immune cell proximity and then exposed to treatments overnight. We first characterized the untreated control condition, representing the baseline immune cell composition and cell states in the RCC TME. Next, we investigated whether a potent T cell activator (anti-CD3/CD28/CD2, ImmunoCult) could effectively stimulate T cells and explored the immunologic effects of PD-1 blockade (pembrolizumab), VEGFR inhibition (VEGFRi; axitinib), and their combination. Treatment effects were evaluated using single-cell RNA sequencing (scRNA-seq) and T cell receptor α/β sequencing (TCRαβ-seq) on sorted CD45^+^ immune cells from eight freshly dissociated tumors. To complement the transcriptomics analyses, an independent batch of cryopreserved tumor dissociates was used for cytometry by time-of-flight (CyTOF) and multiplex cytokine assays, measuring 27 cytokines and growth factors to assess immune cell functionality (a total of nine samples; Fig. 1A). Our study reveals that heterogeneous immune cell contexture leads to selective immune cell activation in the TME of patients with RCC.

## Materials and Methods

### Sex as a biological variable

Our study examined four female and 13 male individuals available to the study. Males are more likely to develop RCC in comparison with females, reflected in our study cohort.

### Patient samples and clinical information

Tumor tissue was collected from 17 patients who underwent nephrectomy at the HUS Helsinki University Hospital (between 2021 and 2023). We included patients undergoing nephrectomy during the study period, with the only exclusion criterion being that patients had to be older than 18 years (mean age = 64 years). Patient clinical information is provided in Supplementary Table S1. The study was conducted in accordance with the Declaration of Helsinki and approved by the Helsinki University Hospital Ethical Committee (Dnro 115/13/03/02/15). All tissue samples and clinical information were obtained after written informed consent.

### Tumor dissociation, *ex vivo* model, and treatments

Primary tumor tissue was transported in MACS Tissue Storage Solution (130-100-008), cleaned of necrotic and fatty tissue, and dissociated using the Miltenyi’s Tumor Dissociation Kit (130-095-929). Cell viability was assessed for all dissociated samples using trypan blue exclusion and quantified with a Countess Automated Cell Counter (Thermo Fisher Scientific, RRID: SCR_020149).

For scRNA + TCRαβ-seq experiments, approximately five million viable cells per sample (around one million cells per treatment condition) were used directly as fresh material. For CyTOF and multiplex cytokine assay, independent subsets of cryopreserved (frozen in 10% DMSO–FBS at −150°C) tumor samples (*n* = 9) were thawed. Fresh (scRNA-seq) or frozen (CyTOF and cytokine multiplexing) single-cell suspensions were embedded in Matrigel (Cultrex Basement Membrane Extract, R&D Systems, cat. #3433-005-01) at approximately 0.5 to 1 × 10^6^ cells/well across an 8-well Nunc Lab-Tek Chamber Slide System, solidified at 37°C, and then incubated overnight prior to addition of treatments. The Cultrex matrix provided a laminin- and collagen-rich three-dimensional (3D) scaffold that supports both immune and nonimmune cell viability and preserves spatial cell–cell and cell–matrix interactions, allowing recovery from dissociation stress and promoting physiologically relevant responses to treatment.

For scRNA + TCRαβ-seq, cells were cultured in RPMI 1640 medium to preserve baseline transcriptional states. For CyTOF and cytokine assays, cells were incubated in AIM V medium (Gibco, 12055091) supplemented with recombinant FGF (100 ng/mL, PeproTech AF-100-18B), EGF (50 ng/mL, PeproTech AF-100-15), IL2 (60 IU/mL, PeproTech 200-02-500UG), sodium pyruvate (10 mmol/L, Merck, S8636-100 mL), B-27 (1.5%, Gibco 17504044), and R-spondin (10% PeproTech 120-38) to support both immune and nonimmune cell viability.

Treatments were added at the following concentrations: VEGFRi, axitinib (300 nmol/L, Pfizer), anti–PD-1, pembrolizumab (50 μg/mL, MedChemExpress, cat. #HY-P9902A), and anti-CD3/CD28/CD2, ImmunoCult (10 μL/mL, StemCell Technologies, cat. #10970). These concentrations were selected based on our published *ex vivo* tumor model studies (18) and manufacturer recommendations to ensure effective target engagement and cell viability under short-term culture conditions. Cells were incubated with treatments at 37°C for 21 to 24 hours. GolgiStop (BD Biosciences, cat. #554724) was added 16 hours before harvest for CyTOF and cytokine multiplexing.

### Antibody labeling and sample preparation

After treatment, Cultrex was dissolved, and the cells were washed and resuspended in PBS + 10% FBS. Cell labeling followed the 10x Genomics Cell Surface Protein Labeling Protocol (CG000149 Rev C) using the workflow for samples with <70% viability, performing two washes.

Cells were blocked with Human TruStain FcX (BioLegend, cat. #422301, RRID: AB_2818986) for 10 minutes at 4°C. Subsequently, 5 μL CD45-APC-H7 antibody (clone 2D1, BD Biosciences, cat. #560178, RRID: AB_1645479) was added to enable CD45^+^ immune cell selection, together with TotalSeq-C anti-human hashtag antibodies (BioLegend cat. #394673, 394675, 394677, 394679, 394683, RRID: AB_2820043, RRID: AB_2820044, RRID: AB_2820045, RRID: AB_2820046, RRID: AB_2904413) recognizing β2-microglobulin and CD298 for multiplexing via cell hashing. Labeled cells were brought to a total volume of 100 μL in chilled PBS + 10% FBS, gently mixed by pipetting, and incubated for 30 minutes at 4°C protected from light. Following labeling, cells were washed twice with 3.5 mL chilled PBS + 10% FBS (300 g, 10 minutes each) and resuspended in chilled PBS for viability staining and flow sorting. After the final wash, cells were resuspended in 250 μL of chilled PBS, viability stains 7AAD (BD Biosciences, cat. #559925, RRID: AB_2869266) and Annexin V (BD Biosciences, cat. #560931) were added, and cells were incubated at 4°C for 15 minutes. Cells were counted using Trypan Blue exclusion on a Countess Automated Cell Counter (Thermo Fisher Scientific, RRID: SCR_020149) and pooled together in equal ratios before being sorted using a BD Influx cell sorter. Viable CD45^+^ lymphocytes and CD45^+^ SSC^high^ cells were collected into a BSA-coated tube, counted, and resuspended at 1,700 to 2,000 cells/μL in PBS with 0.04% BSA.

### Library preparation, sequencing, and data processing

Approximately 33,000 cells from each sample were loaded onto the Chromium Next GEM Chip K using Chromium Next GEM Single Cell 5′ Immune Profiling technology. Gene expression libraries were sequenced using the Illumina NovaSeq 6000 system (RRID: SCR_016387). The scRNA-seq and TCRαβ-seq data were aligned to the human GRCh38 reference genome using 10x Genomics Cell Ranger v6.1.2 (RRID: SCR_016957), with low-quality cells and doublets filtered out. “Cell Ranger multi” generated single-cell feature counts and annotations, whereas “Cellranger aggr” aggregated gene expression and VDJ libraries.

### Multiplexing, demultiplexing, and RNA-seq analysis

Feature-barcode libraries were prepared using 10x Genomics technology, and samples were demultiplexed using the Cell Ranger multi-pipeline.

Raw gene expression and hashtag oligonucleotide (HTO) data were obtained from the sequencing output and processed using the Seurat package in R (v4.0.1; RRID: SCR_016341), with HTO demultiplexing applying centered log-ratio transformation for quality control (QC).

TCR sequencing data were processed using the scRepertoire package, linking TCR data to scRNA-seq barcodes and integrating clonotype information into Seurat objects.

### Data integration, feature selection, and dimensionality reduction

Multiplexed scRNA-seq and TCRαβ-seq data were combined using scvi-tools (v0.13.0; RRID: SCR_026673) and Seurat (v4.1.0; RRID: SCR_016341), with batch effects corrected and visualized using Uniform Manifold Approximation and Projection (UMAP). QC metrics were applied to retain cells with nCount_RNA >1,000, nFeature_RNA >500, and mitochondrial gene expression below 10%. Ribosomal gene expression was quantified using PercentageFeatureSet(). Highly variable features were identified using FindVariableFeatures(), and dimensionality reduction was performed using principal component analysis (PCA) with RunPCA() and UMAP with RunUMAP().

### Clustering, visualization, and reproducibility

After QC metrics were performed, we ended up with 10,003 cells from the control, 12,115 from anti–PD-1, 9,590 from VEGFRi, 10,490 from anti–PD-1 + VEGFRi, and 8,543 cells from anti-CD3/CD2/CD28 treatment conditions. Therefore, we obtained 50,742 cells in total.

The obtained cells were clustered using Seurat functions, annotated based on markers and TCR clonalities, and visualized using ggplot2. The processed data were saved in RDS files to ensure reproducibility.

### Gene set enrichment and transfer learning

Gene set enrichment analysis was performed at the clone level using fgsea (RRID: SCR_020938), identifying expanded clones and conducting differential expression analysis. An scArches transfer learning model mapped single-cell data to a pan-cancer T cell atlas, integrating features using scVI (RRID: SCR_026673).

### Trajectory inference and clonality analysis

To infer developmental lineages in the CD4^+^ and CD8^+^ cell populations, trajectory inference was performed using Slingshot v2.6.0 (RRID: SCR_017012; ref. 19). Manually annotated clusters were provided as input to Slingshot to construct the minimum spanning tree, whereas UMAP embeddings from the data integration step served as the low-dimensional representation. Clustering robustness was evaluated using Totem v0.992 (RRID: SCR_027829; ref. 20). Differentially expressed genes (DEG) among the lineages were identified using a random forest regression model (RRID: SCR_015718; refs. 21, 22). Clonality was assessed using the Gini index.

### Cell–cell communication and pathway analysis

CellChat (v1.6.1 and v2.1.2; RRID: SCR_021946) was used for cell–cell communication analysis of scRNA-seq data, with Seurat-annotated clusters in control condition as input. Each patient was analyzed separately using CellChat’s default settings for secreted and contact signaling. Network centrality scores for sender and receiver signaling were computed using “netP” per patient, and the pathway interaction probabilities were summed per cluster. Aggregated sender and receiver clusters were visualized cohort-wise, with normalized mean values. Treatment-induced communication changes were shown as heatmaps, using median communication probabilities, with missing values imputed as zeroes.

Cell–cell communication analysis for treatment-induced changes was performed using the LIANA+ method (v1.6.1; refs. 23, 24) following the differential contrasts analysis tutorial. Consistent with the tutorial example, the analysis was conducted at a coarse cell annotation level, using CD4^+^ T cells, CD8^+^ T cells, NK cells, monocytes, mast cells, and B cells as cell type labels. This approach helps mitigate issues related to imbalanced cell type abundances across patients and treatments.

To identify the pathways that were upregulated or downregulated in the treatment compared with the control, we first identified the genes that were differentially expressed under treatment condition (in patient–treatment–cluster pseudobulk manner). The lists of these genes were exported and uploaded to Metascape (RRID: SCR_016620), in which hallmark pathway analysis was performed. Pathways enriched in the treatment versus control conditions were identified and categorized based on their functional relevance.

### CyTOF staining

After treatment incubation, 50 μL of DNase I (1 mg/mL; Stemcell Technologies, cat. ##07900) was added to each well and incubated for 15 minutes. The cell suspension was filtered through a 37-μm filter, washed with Maxpar Cell Staining Buffer (CSB; Standard Biotools, cat. #201068-DVS), and centrifuged at 300 × *g* for 5 minutes. The cell pellet was resuspended in 100 μL CSB, with 5 μL Human TruStain FcX (BioLegend, cat. #422301, RRID: AB_2818986) added and incubated at room temperature (RT) for 10 minutes. Dead cells were stained with Rh103 Intercalator (1 μmol/L; Standard Biotools, cat. #PN201103A) for 15 minutes at 37°C. The samples were then stained with CD45 barcoding antibody for 30 minutes, washed, pooled, and stained with surface marker antibodies (Supplementary Table S2). The cells were incubated with Nuclear Antigen Staining Buffer (Standard Biotools, cat. #201068-DVS) for 30 minutes, washed, and stained with an intracellular antibody cocktail for 45 minutes at RT. Finally, the cells were fixed with 1.6% formaldehyde for 10 minutes, centrifuged at 800 × *g* for 5 minutes, and stained overnight at 2°C to 8°C with 125 μmol/L Cell-ID Intercalator-Ir (Standard Biotools, cat. #PN201192B). Samples were acquired on the Helios mass cytometer (RRID: SCR_019916) at the Cell Imaging and Cytometry Core, Turku Bioscience Center.

### CyTOF data analysis

Normalized FCS files were uploaded to Cytobank v10 (Beckman Coulter, RRID: SCR_014043) for data cleanup using the residual, center, offset, viability, DNA1, and DNA3 gates. Treatments were deconvoluted using the following barcoding antibodies: CD45-110Cd (control), CD45-112Cd (pembrolizumab), CD45-114Cd (axitinib), CD45-116Cd (pembrolizumab + axitinib), and CD45-110Cd + CD45-116Cd (ImmunoCult). viSNE clustering was performed on five channels (CD3, CD4, CD8, CD56, and HLA-DR) with equal sampling of ungated events for all CD45^+^ cells, using nine channels for CD8^+^ cells (PD-1, CD39, Ki-67, CD45RO, CCR7, granzyme B, perforin, CD57, and IL7R) and nine channels for CD4^+^ cells (HLA-DR, FOXP3, CD39, Ki-67, CD45RO, CCR7, granzyme B, perforin, and CD57). For treatment response analysis, cells were gated as CD8^+^ (CD8^+^) and CD4^+^ (CD4^+^, HLA-DR-), and differences in the percentage of marker-positive cells (granzyme B, perforin, IFN-γ, TNF, CCL4, Ki-67, and IL2) between the treatment and control groups were calculated.

### Cytokine multiplex analysis

Before CyTOF staining, 200 μL of cell culture medium was collected from each well and stored at −70°C for cytokine analysis and then defrosted on the day of analysis. Media samples from *ex vivo* treatments were analyzed for 27 cytokines, chemokines, and growth factors using a customized Bio-Plex Pro Human Cytokine Screening Panel, 27-plex (Bio-Rad, cat. ##M500KCAF0Y, RRID: AB_2893118). The panel was customized by replacing four panel analytes (bFGF, G-CSF, PDGF-BB, and IL1ra) with IL1α, MIF, SDF1-α, and TNF-β. Samples were prepared per the manufacturer’s instructions, with a 20x dilution of custom analytes. Data were acquired using a Luminex 200 (RRID: SCR_018025), with cytokine concentrations extrapolated from standard measurements. Values below the standards were set at 0.5 × the lowest standard, and high values were estimated using software. Treatment responses were calculated as “effect size” using Hedges’ g and interpreted as “medium” (0.5) and “large” (0.8) effects. Individual patient responses were assessed by calculating the differences in cytokine concentrations between the treatment and control groups.

### NanoString gene expression profiling

The patient-derived *ex vivo* model was established for two patients. For each patient, freshly isolated dissociate and frozen dissociated was used for the model, and anti-CD3/CD28/CD2 (Immunocult) treatment was applied for either 24 hours or 48 hours. Following treatment, total RNA was isolated using RNeasy Kit (Qiagen), and DNA removal was performed after RNA isolation (Zymo research) according to the manufacturer’s instructions. All samples went through QC (Qubit) before gene expression analysis with the NanoString nCounter gene expression platform (NanoString Technologies). nCounter Human PanCancer Immune Profiling Panel consisting of 770 immune-related genes. Per sample, 50 ng of total RNA in a final volume of 5 μL was mixed with a reporter codeset, hybridization buffer, and capture codeset. Samples were hybridized overnight at 65°C for 20 hours. Hybridized samples were run on the NanoString nCounter SPRINT profiler. NanoString analysis was carried out at the functional genomics unit at the University of Helsinki.

### Statistical analysis

Statistical methods were applied to verify the results. Values for *N* (sample size), P (*P* value), and specific statistical tests performed for each experiment are included in the appropriate figure legend or main text. Paired *t* tests, Bonferroni correction, and effect size calculations were conducted to assess treatment responses. A two-sided Wilcoxon rank-sum test was used when comparing treatment with control.

## Results

### Single-cell profiling of RCC tumor-immune microenvironment

Tumor tissues were collected from 17 treatment-naïve patients with localized RCC during surgical resections (Fig. 1A; Table 1; Supplementary Table S1). Ten tumors were classified as ccRCC, whereas the remaining cases included papillary RCC (*n* = 3), oncocytoma (*n* = 2), chromophobe RCC (*n* = 1), and one tumor of unclassified phenotype (*n* = 1; Fig. 1B). Necrotic tissue was observed in five samples, but no metastases were observed. Tumor size varied, with 11 patients having tumors smaller than 7 cm (pT1a: *n* = 6; pT1b: *n* = 4; unknown stage: *n* = 1), whereas six patients had larger tumors (pT2a: *n* = 2, pT3a: *n* = 4; Fig. 1B).

**Table 1.** Sample cohort and patient characteristics.

Patients	*n*
Age in years: mean (range)	65 (32–81)
Gender: *n* (%)	​
Male	13 (76.5)
Female	4 (23.5)
Histology: *n* (%)	​
Clear cell	10 (58.8)
Chromophobe	1 (5.9)
Oncocytoma	2 (11.8)
Papillary	3 (17.6)
Unclassified	1 (5.9)
Tumor, node, metastasis stage pT-class at the time of diagnosis: *n* (%)	​
pT1a	6 (35.3)
pT1b	4 (23.5)
pT2a	2 (11.8)
pT3a	4 (23.5)
Unknown	1 (5.9)
Tumor, node, metastasis invasion at the time of diagnosis: *n* (%)	​
pNX	3 (17.65)
Fat/vena renalis	3 (17.65)
None	11 (64.7)

To model the RCC TME in 3D *ex vivo*, tumor dissociates were embedded in an ECM to maintain the spatial proximity of the tumor and immune cells and prevent immune cell migration into the surrounding media. We confirmed that the model preserved the composition and viability of the main tumor-infiltrating immune subsets, including T and NK cells, during *ex vivo* culture (Supplementary Fig. S1A and S1B), providing a baseline for subsequent analyses. To further characterize the immune contexture of the RCC TME, we performed detailed phenotyping of infiltrated immune cells using scRNA + TCRαβ-seq (*n* = 8) on freshly dissociated tumors and mass cytometry (CyTOF; *n* = 9) on separate cryopreserved tumor dissociates.

From the explants, we flow sorted and sequenced CD45^+^ immune cells: lymphocytes and myeloid cells. After QC, our scRNA + TCRαβ-seq dataset included 10,003 immune cells, capturing a comprehensive representation of the immune cell types within the untreated control TME, representing our baseline. We performed batch correction at the patient–treatment level to minimize possible batch effects and enable joint analysis. Using known lineage markers, we identified T cells (*CD3E*, *CD3D*, *CD8A*, and *CD4*) and NK cells (*GNLY*, *KLRD1*, and *NKG7*) as the most abundant infiltrating immune cell types among the lymphocytes (Fig. 1C and D; Supplementary Fig. S1C and S1D). Each patient also harbored antigen-presenting cells (APC) within their TME, mostly B cells (*MS4A1*, *CD79A*, and *CD79B*) and monocytes (*CD14* and *CD68*). Additionally, a distinct population of mast cells (*CPA3*, *MS4A2*, and *TPSAB1*) was identified (Fig. 1C and D).

To complement the scRNA + TCRαβ-seq analysis, mass cytometry was performed with nine additional tumor samples (Fig. 1A; Supplementary Table S2). CyTOF data confirmed a high infiltration of T cells (CD45^+^, CD3^+^, CD8^+^ and CD45^+^, CD3^+^, CD4^+^) and NK cells (CD45^+^, CD3^−^, CD56^+^) and identified APCs (CD45^+^, CD3^−^, CD56^−^, HLA-DR+) as one of the main immune cell populations (Fig. 1E; Supplementary Fig. S1E–S1G).

### CD8^+^ T cell diversity within the RCC TME

**Figure 2.**
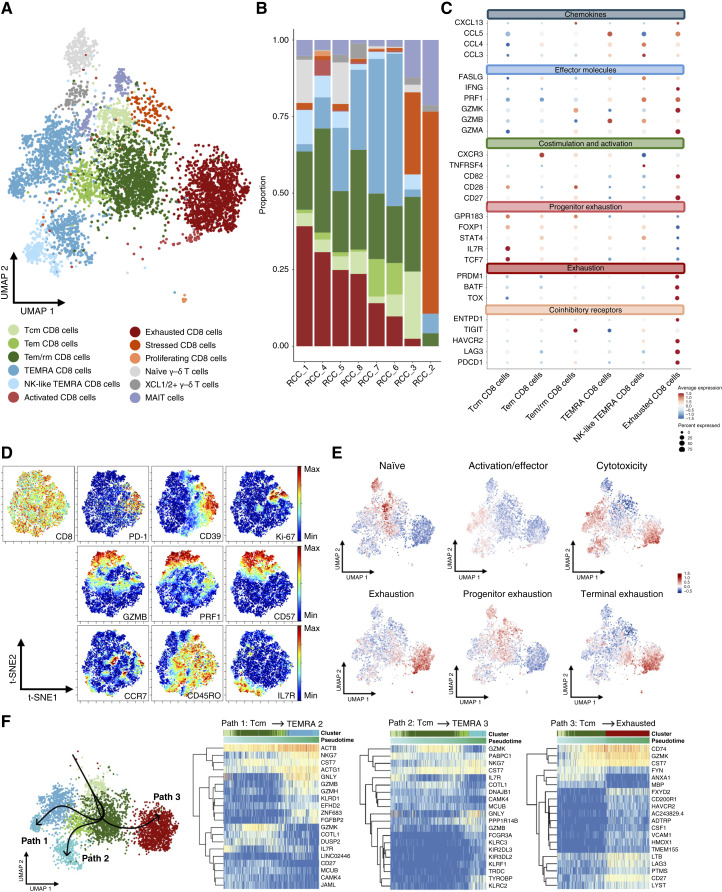
Characterization of CD8^+^ T cells in control. **A,** UMAP of 5,177 CD8^+^ T cells in untreated control condition, captured across eight patient samples (RCC_1–RCC_8), colored by characterized cell subtypes. **B,** Bar plot showing distribution of characterized CD8^+^ cell subtypes across all eight patients, ordered by the proportion of exhausted CD8^+^ cells in each patient. **C,** Dot plot showing average expression of selected genes across identified cell clusters. Each dot represents a specific gene–cluster combination, in which the size of the dot indicates the percentage of cells within the cluster expressing the gene and the color intensity reflects the average expression level of the gene in those cells. **D,** CyTOF analysis of CD8^+^ T cells in control condition from nine patients (RCC_9–RCC_17). Marker expression patterns are visualized using t-distributed stochastic neighbor embedding (t-SNE) plots. **E,** Signature score for naïve, activation/effector, cytotoxicity, exhaustion, progenitor exhaustion, and terminal exhaustion CD8^+^ T cell signatures plotted on CD8^+^ T cell UMAP. **F,** UMAP depicting the pseudotime inference of CD8^+^ T cells, showing three distinct paths. Heatmaps on the right show the top 20 genes that change their expression over the course of path progression.

We subclustered CD8^+^ T cells in the scRNA + TCRαβ-seq data into 12 clusters, characterized by markers and feature signatures indicative of cell lineage and function (Fig. 2A; Supplementary Fig. S2A and S2B; Supplementary Table S3). Exhausted CD8^+^ cells, terminally differentiated effector memory (TEMRA) CD8^+^ cells, and effector memory/resident memory cells (Tem/rm) CD8^+^ cells were the most abundant subclusters (Fig. 2B and C; Supplementary Fig. S2C).

The exhausted CD8^+^ T cell cluster exhibited high expression of *TOX*, *BATF*, and *PRDM1*, transcription factors critical for T cell exhaustion (25–27), along with elevated expression of several co-inhibitory checkpoint molecules [*PDCD1* (PD-1), *LAG3*, *HAVCR2* (TIM3), and *ENTPD1* (CD39)], and genes related to cytolytic activity (e.g., *GZMA*, *GZMB*, *GZMK*, and *PRF1*), *TNFRSF9* (4-1BB), and *CXCL13*, suggesting antigen experience (25, 28). CyTOF analysis confirmed a cell state co-expressing high levels of PD-1 and CD39 with moderate levels of cytotoxicity-related proteins, along with a distinct cytotoxic (GZMB+, PRF1+, CD57^+^) population (Fig. 2D). Interestingly, the proliferation marker Ki-67 staining was partially enriched in exhausted cells. In addition, we identified a small proliferative cluster marked by *MKI67* and *TOP2* in our scRNA-seq data (Supplementary Fig. S2A–S2C). This finding is consistent with previous reports in RCC demonstrating that proliferation can persist in exhausted CD8^+^ T cells, although at a reduced level (15). Notably, 43% (6 of 14) of the T cell clones within this cluster overlapped with the exhausted cluster, suggesting that these cells may be in a preexhausted state, retaining some proliferative capacity despite their progression toward exhaustion (Supplementary Fig. S2D).

The largest CD8^+^ cluster, comprising approximately one-fourth of the cytotoxic T cell population, expressed effector [*TBX21* (T-BET), *GZMH*, *KLRD1*, *KLRG1*, *FGFBP2*, and *FCGR3A* (CD16a)] and cytolytic activity (*PRF1* and *GZMB*) markers characteristic of TEMRA T cells. This observation aligns with previous pan-cancer scRNA-seq data analyses, which reported an unusually high proportion of infiltrating TEMRA cells in RCC (25). We also identified a related cluster expressing NK cell markers (*GNLY* and *TYROBP*), designated as NK-like TEMRA cells (Supplementary Fig. S2A–S2C).

Three memory-associated CD8^+^ T cell clusters were identified: central memory T cells (Tcm; *TCF7*, *IL7R*, *NELL2*, *SELL*, and *CCR7*), Tem cells (*GZMH* and *GZMM*), and Tem/Trm cells (*CXCR6*, *ITM2A*, and *ITM2C*). The RCC TME also harbored low-frequency unconventional T cell subsets, MAIT cells (*TRAV1-2* and *KLRB1*), and γ–δ T cells (*TRDV1*, *TRDV3*, and *TRGC1*; Supplementary Fig. S2A–S2C).

Having established CD8^+^ T cell clusters, we correlated them with previously identified pan-cancer T cell states (28). We projected naïve, activator/effector, cytotoxicity, and exhaustion signatures on CD8^+^ T cell UMAP, confirming the expected signature enrichment and overlap of cytotoxicity with both activated and exhausted cell states (Fig. 2E; Supplementary Fig. S2E).

We further identified cells resembling a progenitor-exhausted state, which has previously been associated with responses to immune checkpoint blockade (Fig. 2C and E; and Supplementary Tables S4 and S5; ref. 29). Scoring individual cells for progenitor and exhaustion gene signatures (16, 29) revealed elevated progenitor-exhaustion score in stressed cells, whereas terminal exhaustion score was higher in activated, exhausted, and proliferating CD8^+^ subsets. These findings align with prior studies in melanoma and RCC, demonstrating the persistence of progenitor-exhausted and distinct exhausted CD8^+^ populations in the RCC TME (16, 29).

Pseudotime trajectory analysis on the most abundant cell CD8^+^ clusters revealed three distinct pathways (Fig. 2F). Two trajectories progressed from Tcm cells toward TEMRA clusters (TEMRA 2 and TEMRA 3), with intermediate states transitioning to effector cell states indicative of cytolytic activity and NK-like profiles (Supplementary Fig. S2B). The third trajectory culminated in exhaustion and was marked by downregulation of early/resting genes (*ANXA1*, *FYN*, and *MBP*) and gradual upregulation of dysfunction-related genes (*LAG3* and *HAVCR2*).

### The RCC TME displays heterogeneous CD4^+^ T cell states

**Figure 3.**
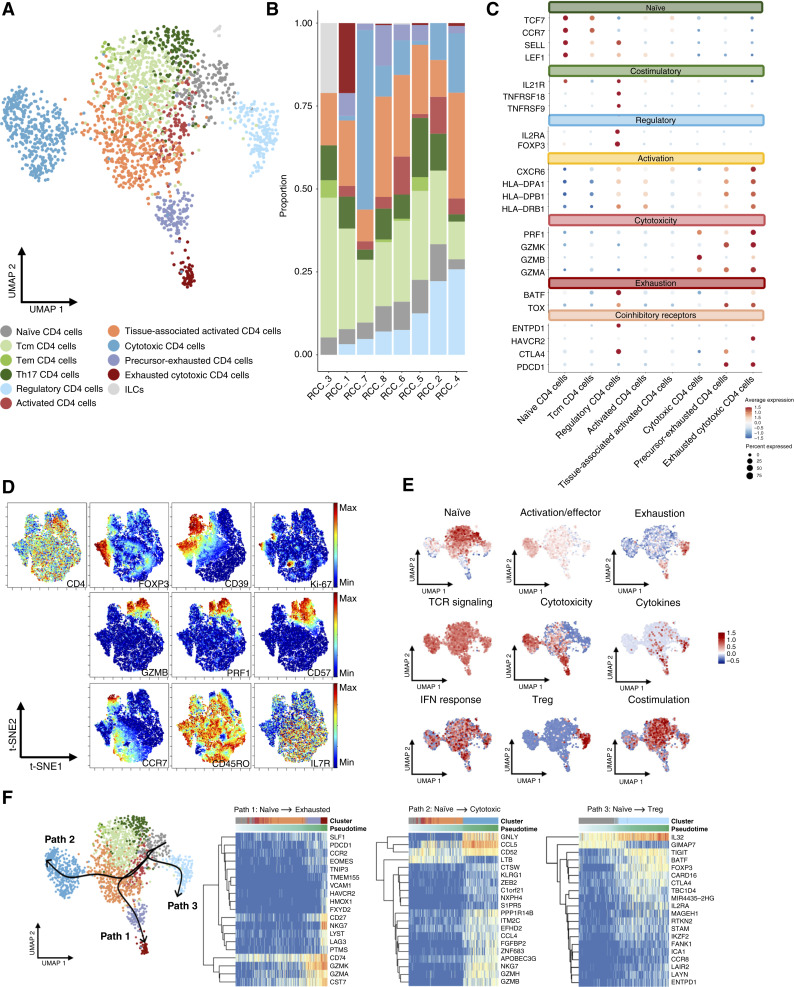
Characterization of CD4^+^ T cells in control. **A,** UMAP of 2,136 CD4^+^ T cells in untreated control condition captured across eight patient samples (RCC_1–RCC_8), colored by characterized cell types. **B,** Bar plot showing distribution of characterized CD4^+^ cell types across all eight patients, ordered by the proportion of regulatory CD4^+^ cells in each patient. **C,** Dot plot showing average expression of selected genes across identified cell clusters. Each dot represents a specific gene–cluster combination, in which the size of the dot indicates the percentage of cells within the cluster expressing the gene, and the color intensity reflects the average expression level of the gene in those cells. **D,** CyTOF analysis of CD4^+^ T cells in control condition from nine patients (RCC_9–RCC_17). Marker expression patterns are visualized using t-distributed stochastic neighbor embedding (t-SNE) plots. **E,** Signature score for naïve, activation/effector, exhaustion, TCR signaling, cytotoxicity, cytokines, IFN response, Treg, and co-stimulation CD4^+^ T cell signatures plotted on CD4^+^ T cell UMAP. **F,** UMAP depicting the pseudotime inference of CD4^+^ T cells, showing three distinct paths. Heatmaps on the right show the top 20 genes that change their expression over the course of path progression.

CD4^+^ T cells participate in tumor immunity by modulating the TME and, in some cases, may even exert direct cytolytic activity (30). We identified 12 phenotypically distinct CD4^+^ T cell clusters, revealing diverse cellular states complementing our findings for CD8^+^ T cells (Fig. 3A–C; Supplementary Fig. S3A; Supplementary Table S6). As expected, naïve (*LEF1*, *SELL*, *SOCS3*, *CCR7*, and *TCF7*), Tcm (*S1PR1*, *ICAM2*, and *KLF2*), Tem (*NEAF1*, *ANXA2*, and *FTH1*), T helper 17 (Th17; *CCR6*, *RORA*, and *RORC*), and activated (*HLA-DPA1*, *HLA-DPB1*, and *HLA-DRB1*) CD4^+^ T cells were identified as abundant cell states (Fig. 3A; Supplementary Fig. S3A). A regulatory CD4^+^ (Treg) cluster was marked by *FOXP3*, *IL2RA*, and *CTLA4*, together with high expression of costimulatory molecules and cytokine receptors (*TNFRSF9*, *TNFRSF18*, and *IL21R*), suggesting activation. CyTOF analysis confirmed Treg accumulation (CD3^+^, CD4^+^, FOXP3+) in the TME and revealed high expression of CD39 (*ENTPD1*), indicating a highly active Treg subset modulating an immunosuppressive TME (Fig. 3D; refs. 31–33).

Two CD4^+^ clusters were identified as exhausted, exhibiting high expression of *TOX* and variable expression of checkpoint molecules, (*PDCD1*, *CTLA4*, and *HAVCR2*), along with cytotoxicity-associated genes (*GZMA*, *PRF1*, and *GZMK*), and co-stimulatory molecules (*CD27* and *CD28*; Fig. 3A–C; Supplementary Fig. S3A and S3B). This implies that these cells may remain engaged in antitumor responses while being constrained by exhaustion mechanisms, similar to exhausted CD8^+^ T cells (34). One CD4^+^ T cell cluster exhibited high expression of cytotoxic genes (*GZMA*, *GZMB*, *GZMH*, *PRF1*, and *GNLY*; Fig. 3A–C; Supplementary Fig. S3A and S3B) and was annotated as cytotoxic CD4 according to previously identified similar marker expression profile (28, 30). This cluster was predominantly composed of cells from a patient with high frequency of cytotoxic CD8^+^ T cells (Figs. 2B and 3B). Cytotoxic CD4^+^ cells (GZMB+, PRF1+, CD57^+^) were also observed in the CyTOF analysis (Fig. 3D). Our manual annotation of CD4^+^ T cells was supported by published curated gene signatures (Fig. 3E; Supplementary Table S7; ref. 28).

Pseudotime trajectory analysis showed CD4^+^ T cells progressing from naïve to exhausted, cytotoxic, and regulatory lineages. Two trajectories advanced from naïve to cytotoxic, marked by increased expression of granzymes (*GZMA*, *GZMK*, *GZMB*, and *GZMH*), with one trajectory additionally exhibiting exhaustion markers such as *LAG3*. A third trajectory indicated a shift from a naïve to a regulatory phenotype, characterized by increased expression of *FOXP3*, *CTLA4*, and *IL2RA* (Fig. 3F).

### CD56 bright and CD56 dim cell subsets of the TME

NK cells constituted the second most abundant immune cell population after T cells. Seven distinct NK clusters were identified, including three CD56 bright and four CD56 dim subsets (Supplementary Fig. S3C–S3F; Supplementary Table S8). CD56 bright clusters were linked to quiescent cell state (*NCAM1*, *KLRC1*, and *KLRB1*), tissue residency (*ITGAE*, *ITGA1*, and *CXCR6*), or early differentiation stage (*KIT*, *IL7R*, *IL1R1*, *TCF7*, and *SELL*), indicating potential for further differentiation (Supplementary Fig. S3C–S3F).

CD56 dim NK cells exhibited either an activated state (*CXCR3*, *CXCR4*, and *JUN*) or IFN-I responsive state (*MX1-2* and *OAS1-3*). Cytotoxic NK cells showed potent cytolytic activity (*KIRs*, *KLF2*, *GZMH*, *GZMB*, and *PRF1*), indicating their capacity to directly kill tumor cells via granzyme- and perforin-mediated cytotoxicity. Lastly, we identified a small cluster with high expression of cytokines, including *CCL3*, *CCL4*, *TNF*, and *IFN-*γ (Supplementary Fig. S3C and S3F).

In addition to NK cells, we identified myeloid population representing a heterogenous compartment that included mast cells (*TSPAB1* and *CPA3*), inflammatory monocytes (*IL1B* and *HMOX1*), and intermediate monocytes (*LYZ* and *FCGR3A*; Supplementary Fig. S3G and S3H). B-cell population consisted of naïve and class-switched memory differentiated states. Naïve subset expressed higher levels of *TLC1A*, *IGHM*, and *IGHD*, whereas memory B cells were enriched for *IGHG1*, *IGHG2*, and *S100A4* (Supplementary Fig. S3I and S3J).

### Immunologic response of T cells to potent T cell stimulation within the RCC TME

Our immune cell annotation suggested that the patient-derived culture model retains the previously described CD8^+^ and CD4^+^ T cell TME subtypes and their cellular states (15, 25, 28), as well as patient heterogeneity. We then explored whether the model could simulate immunologic responses within the TME *ex vivo*. Using fresh and frozen tumor samples, we assessed cytokine and growth factor secretion into the culture media at the control (Supplementary Fig. S4A) and with two pilot cases, 24 and 48 hours after anti-CD3/CD28/CD2 treatment. Concordant immune cell activation was observed (Supplementary Fig. S4B–S4F), allowing us to utilize this model to analyze early immune responses in detail (18).

**Figure 4.**
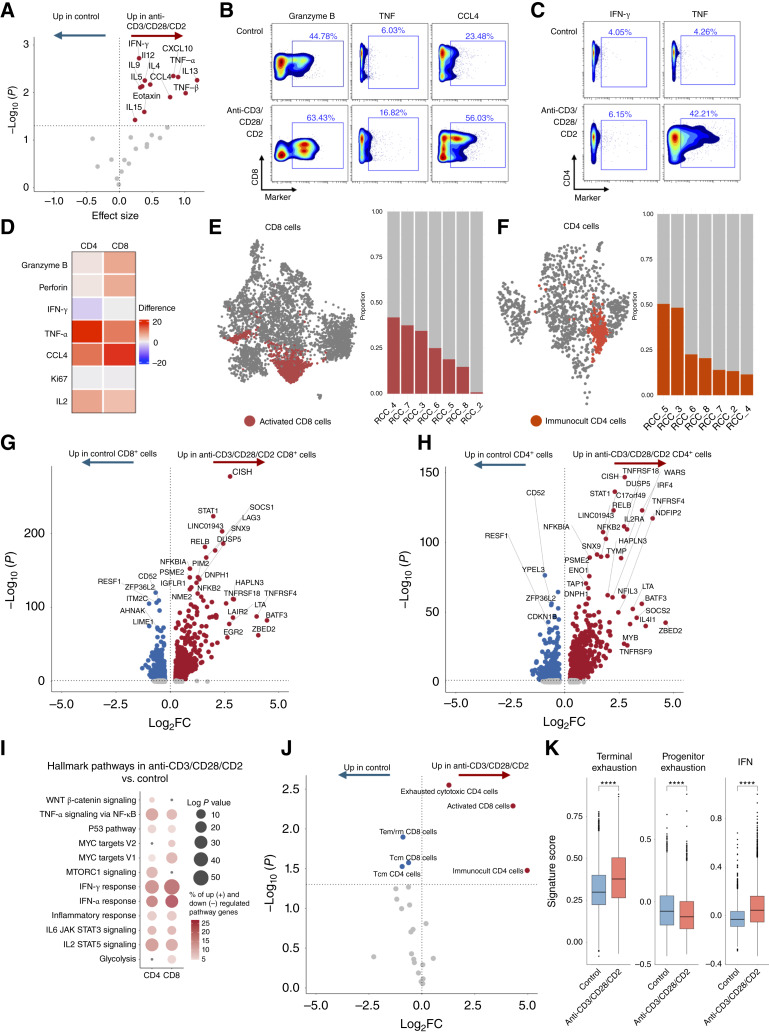
Characterization of the effects of a strong T cell activator, ImmunoCult (anti-CD3/CD28/CD2), on the RCC TME. **A,** Volcano plot illustrating changes in cytokine secretion in response to anti-CD3/CD28/CD2. Each point represents a cytokine, and statistically significantly upregulated cytokines are annotated and highlighted in dark red. **B** and **C,** CyTOF analysis for comparison of granzyme B, TNF (TNF-α), CCL4, or INF-γ and TNF expression between control and anti-CD3/CD28/CD2 in CD8^+^ (**B**) and CD4^+^ (**C**) T cells, respectively. Gated populations depict the percentage of positive cells for each marker. **D,** Heatmap showing changes in the percentage of marker-positive cells between control and anti-CD3/CD28/CD2 conditions in CD8^+^ and CD4^+^ T cells. **E,** UMAP of CD8^+^ T cells highlighting a newly formed “activated CD8 cell” cluster in response to anti-CD3/CD28/CD2 treatment. Bar plot to the right shows the proportion this newly formed cluster occupies in CD8^+^ T cells of each of the eight patients in anti-CD3/CD28/CD2 treatment. **F,** UMAP of CD4^+^ T cells highlighting a newly formed “ImmunoCult CD4 cells” cluster in response to anti-CD3/CD28/CD2 treatment. Bar plot to the right shows the proportion this newly formed cluster occupies in CD4^+^ T cells of each of the eight patients in anti-CD3/CD28/CD2 treatment. **G** and **H,** Volcano plot illustrating changes in gene expression in response to anti-CD3/CD28/CD2 in CD8^+^ T cells (**G**) or CD4^+^ T cells (**H**). Each point represents a gene, the significantly upregulated genes are colored dark red, whereas downregulated genes are dark blue. Only significantly regulated genes are labeled [adjusted *P* value < 0.05 and abs(avg_log_2_FC) > 0.5]. **I,** Dot plot of selected immunology-related hallmark pathways, comparing anti-CD3/CD28/CD2 and control conditions. Genes were identified using the FindMarkers method and included if the *P* value was < 0.05. Circle size represents the log*P* value, whereas color indicates the percentage of genes in our dataset that are upregulated (+) (in red) or downregulated (−) (in blue). **J,** Volcano plot illustrating changes in cluster size in response to anti-CD3/CD28/CD2. Each point represents a T cell cluster (CD8^+^ T cells and CD4^+^ T cells). Cell populations that are enriched in anti-CD3/CD28/CD2 and statistically significant (adjusted *P* value < 0.05 and log_2_FC > 0) are highlighted in dark red, and cell populations that are diminished in anti-CD3/CD28/CD2 and statistically significant (adjusted *P* value < 0.05 and log_2_FC < 0) are colored in dark blue. FC, fold change. **K,** Signature score distributions for terminally exhausted, progenitor exhausted, and IFN CD8^+^ T cell signatures comparing control and anti-CD3/CD28/CD2 cells. Statistical significance of differential signature enrichment (*P* value) between subtypes was determined by two-sided Wilcoxon rank-sum test (****, *P* ≤ 0.0001).

Next, we investigated the immunotherapy responses of 17 patients with RCC. To better understand early immune reactions, we studied immune cells triggered by a potent T cell activator (anti-CD3/CD28/CD2, ImmunoCult) using cytokine profiling, CyTOF, and scRNA + TCRαβ-seq. Cytokine profiling revealed robust immune activation in the TME of each studied patient, with significant inflammatory interleukin, chemokine, TNF, and INF-γ responses (Fig. 4A; Supplementary Fig. S4G and S4H; Supplementary Table S9). To understand the T cell subtypes driving this response, we further assessed their stimulation by CyTOF. Both CD8^+^ and CD4^+^ T cells contributed to the response, as detected by the increase in effector cytokine TNF (TNF-α)- and chemokine (CCL4)-producing cells (Fig. 4B–D). INF-γ was primarily expressed by T cells, NK cells, and monocytes, however showing variable response in individual patients T cells, likely reflecting differences in the activation kinetics across individual tumors (Supplementary Fig. S4I–S4L). Additionally, cytotoxic cells did not exhibit a marked increase in their cytolytic potential (Supplementary Fig. S4I). In scRNA-seq analysis, introduction of the T cell activator into the TME led to the emergence of a distinct cell cluster in both CD8^+^ and CD4^+^ T cells. Cells within these new clusters comprised up to 50% of the anti-CD3/CD28/CD2–treated T cell populations (Fig. 4E and F). Both the newly formed CD8^+^ and CD4^+^ T cell populations displayed an activated phenotype, marked by the expression of *TNFRSF4*, *TNFRSF18*, and *BATF3* in CD8^+^ cells and *IL2RA*, *TNFRSF4*, *IRF4*, and *BATF3* in CD4^+^ cells (Fig. 4G and H). Furthermore, these populations showed enrichment in TNF signaling via NF-κB, IL2 signaling, and type I and II IFN responses (Fig. 4I; Supplementary Fig. S4M), and this response was also evident in the other immune cell types (Supplementary Fig. S4N; Supplementary Tables S10–S15). Taken together, these results indicate robust immune activation and effector function within the TME.

Subsequently, we sought to determine the cell states giving rise to the activated cell populations. We compared relative cell proportions across clusters between control and immune activator–treated conditions. CD8^+^ Tem/Trm and Tcm clusters were less abundant following activation, suggesting that these cells contributed to the activated CD8^+^ state (Fig. 4J). Concurrently, we observed a transition from CD4^+^ Tcm cells toward exhausted cytotoxic CD4^+^ cells and ImmunoCult CD4^+^ clusters (Fig. 4J). Finally, we noted that immune activation induced higher overall exhaustion and lower progenitor-exhaustion states in CD8^+^ cells, as well as an induced IFN signature (Fig. 4K; Supplementary Fig. S4O; Supplementary Table S5). These findings suggest that treatment with a strong immune activator promotes dynamic reshaping of the T cell landscape, enhancing activation potential while reducing memory-associated phenotypes.

### Immunologic response of T cells to anti–PD-1 and VEGFRi within the RCC TME

**Figure 5.**
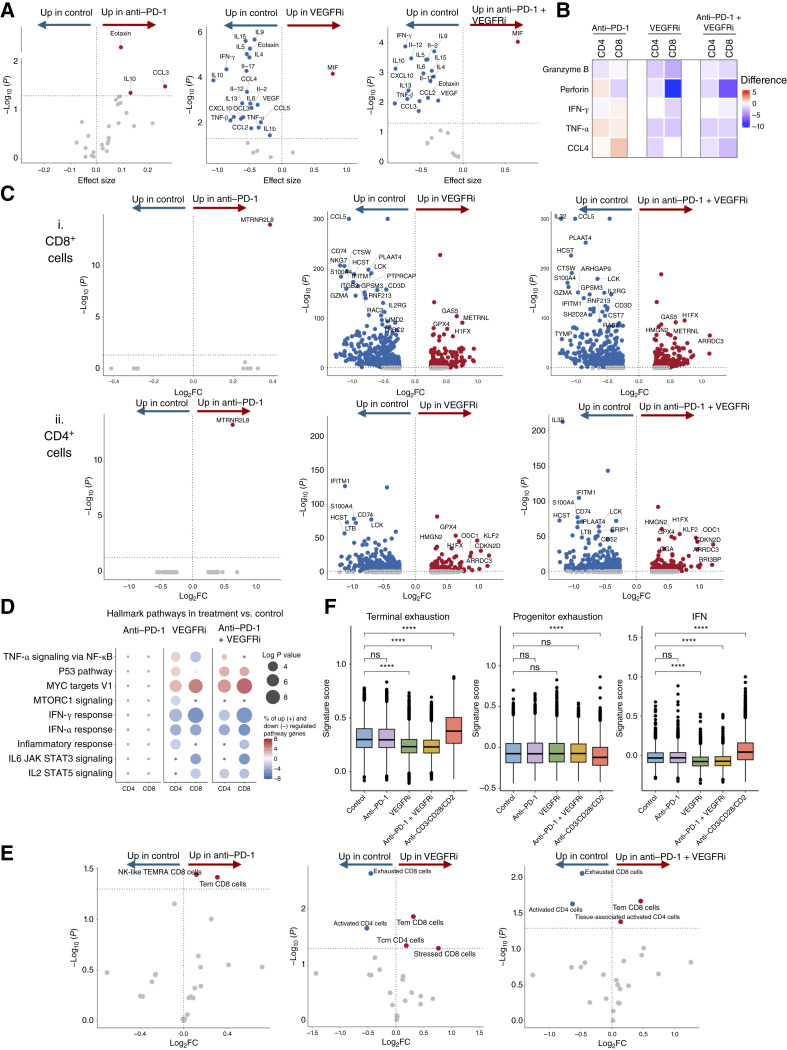
The effects of anti–PD-1 (pembrolizumab), VEGFRi (axitinib), and their combination on immune cell functionality in the RCC TME. **A,** Volcano plot illustrating changes in cytokine secretion in response to anti–PD-1, VEGFRi, or anti–PD-1 + VEGFRi combination. Each point represents a cytokine, and upregulated and statistically significant cytokines are highlighted in dark red, whereas downregulated and statistically significant cytokines are colored in dark blue [−log_10_(P) > −log_10_(0.05) or *P* > 0.05]. **B,** Heatmap comparing change in immune effector markers expression between control, anti–PD-1, VEGFRi, or anti–PD-1 + VEGFRi combination in CD8^+^ and CD4^+^ T cells. **C,** Volcano plot illustrating changes in gene expression in response to anti–PD-1, VEGFRi, or anti–PD-1 + VEGFRi combination in CD8^+^ T cells (i) or CD4^+^ T cells (ii). Each point represents a gene, the upregulated genes are colored dark red, whereas downregulated genes are dark blue. Only significant genes are labeled [adjusted *P* value < 0.05 and abs(avg_log_2_FC) > 0.5, and in the case of anti–PD-1 adjusted *P* value < 0.05 and abs(avg_log_2_FC) > 0.3]. **D,** Dot plot of selected immunology-related hallmark pathways, comparing anti–PD-1, VEGFRi, or anti–PD-1 + VEGFRi to control condition. Genes were identified using the FindMarkers method and included if the *P* value was < 0.05. Circle size represents the log*P* value, whereas color indicates the percentage of genes in our dataset that are upregulated (+) or downregulated (−). **E,** Volcano plot illustrating changes in relative cluster size in response to anti–PD-1, VEGFRi, or anti–PD-1 + VEGFRi combination treatments. Each point represents a T cell cluster (CD8^+^ T cells and CD4^+^ T cells). Cell populations that are enriched in treatment and statistically significant (adjusted *P* value < 0.05 and log_2_FC > 0) are highlighted in dark red, and cell populations that are diminished in treatments and statistically significant (adjusted *P* value < 0.05 and log_2_FC < 0) are colored in dark blue. FC, fold change. **F,** Signature score distributions for terminally exhausted, progenitor exhausted and IFN CD8^+^ T cell signatures comparing control with anti–PD-1, VEGFRi, anti–PD-1 + VEGFRi, and anti-CD3/CD28/CD2. Statistical significance of differential signature enrichment (*P* value) between subtypes was determined by two-sided Wilcoxon rank-sum test (ns, *P* > 0.05; ****, *P* ≤ 0.0001).

We next explored the immunologic effects of clinically relevant treatments in the RCC TME. Given the known sensitivity of RCC to checkpoint inhibitors and antiangiogenic agents, patient-derived *ex vivo* cultures were treated with a checkpoint inhibitor (anti–PD-1, pembrolizumab), a VEGFRi (axitinib), or their combination. Initial profiling of 27 secreted cytokines indicated modest immune activation with anti–PD-1 alone, characterized by significantly increased release of CCL3, eotaxin, and IL10 (Fig. 5A; Supplementary Fig. S5A; Supplementary Table S9). In contrast, VEGFRi led to a marked decrease in the expression of multiple cytokines, and the combination treatment largely mirrored the VEGFRi response (Fig. 5A; Supplementary Fig. S5A; Supplementary Table S9). Consistent with these findings, both CD8^+^ and CD4^+^ T cells exhibited downregulation of effector and cytolytic molecules in the CyTOF data following VEGFRi treatment (Fig. 5B). Whereas PD-1 blockade induced stimulation in some individuals, the strength of the response remained modest compared with anti-CD3/CD28/CD2 activation (Fig. 5B; Supplementary Fig. S5B and S5C).

To investigate whether the effects of the VEGFRi could be attributed to VEGF–VEGFR signaling within immune cells, we further explored the control scRNA-seq data, representing our baseline for the expression of VEGF ligands and receptors. We observed no detectable expression of *KDR* (VEGFR2) and only sparse expression of *FLT1* (VEGFR1) and *FLT4* (VEGFR3), which were largely restricted to the monocyte population. In contrast, VEGF ligand expression was mainly detected in mast cells (Supplementary Fig. S5D), suggesting that any immunomodulatory effects of VEGFRi on T cells may be secondary.

Transcriptional profiling further supported these observations. Anti–PD-1 did not significantly alter transcription across T cells and other immune cell subtypes, whereas VEGFRi and its combination with anti–PD-1 downregulated various genes (Fig. 5C), particularly within the IFN, IL2–STAT5, and IL6–JAK–STAT3 signaling pathways (Fig. 5D; Supplementary Fig. S5E–S5H; Supplementary Tables S10–S15). This effect was evident in both T cells and NK cells but was most pronounced in CD8^+^ T cells. Pathway analysis further demonstrated that VEGFRi downregulated IFN-α/γ, IL2–STAT5, TNF-α via NF-κB, and IL6–JAK–STAT3 pathways (Fig. 5D; Supplementary Fig. S5E–S5H; Supplementary Tables S10–S15), underscoring a reduction in proinflammatory signaling. In the combination treatment, transcriptional responses were largely driven by VEGFRi.

Analysis of relative T cell phenotype abundance in response to treatments revealed that PD-1 blockade led to a modest increase in Tem and NK-like TEMRA cells, indicating a slight shift toward memory and cytotoxic phenotypes (Fig. 5E). In contrast, VEGFRi treatment induced changes within both the CD8^+^ and CD4^+^ subpopulations. CD8^+^ T cells exhibited a reduction in exhausted subsets accompanied by an increase in Tem and stressed cells, suggesting an enhanced memory phenotype, but also heightened cellular stress, possibly due to TME pressures or VEGFRi itself. Among CD4^+^ T cells, axitinib induced a “shift” from activated to Tcm cells, indicative of a phenotype favoring rapid reactivation and long-term survival.

We further examined the effect of anti–PD-1 and VEGFRi on progenitor-exhausted and exhausted cell states within CD8^+^ T cells. VEGFRi and combination treatments reduced CD8^+^ T cell exhaustion across patients (Fig. 5F; Supplementary Fig. S5I; Supplementary Table S5). Conversely, progenitor exhaustion status remained unaltered by treatments relative to the control. Consistent with the reduced cytokine secretion, VEGFRi-treated cells displayed lower IFN signaling scores than the control (Fig. 5F; Supplementary Fig. S5I and S5J).

### Expanded CD8^+^ T cell clones display different activation dependent on their cellular state

To capture treatment responses beyond transcriptional changes, we characterized T cells for their TCRs and clonal expansion. We identified 4,966 unique clonotypes across both CD8^+^ and CD4^+^ T cells across all patients, with each clonotype representing a distinct TCR sequence corresponding to an individual T cell clone.

**Figure 6.**
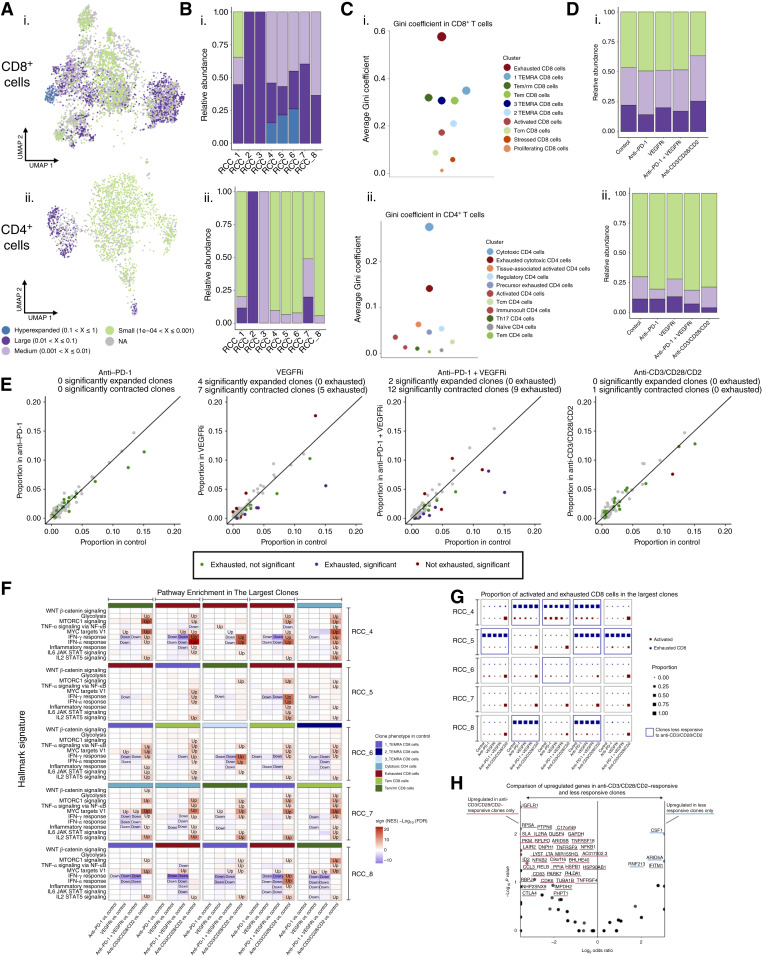
Differential activation of T cell clones dependent on their prior cell state. **A,** The UMAP view of TCR distribution displaying clonotype frequency in CD8^+^ (i) and CD4^+^ (ii) T cell populations. **B,** Bar plot showing the relative abundance of clones among patients with RCC (RCC_1–RCC_8) in CD8^+^ (i) and CD4^+^ (ii) T cells. **C,** Average clonality of TCR repertoires at the cluster level, shown separately for CD8^+^ (i) and CD4^+^ (ii) T cells. **D,** Bar plot showing the relative abundance of clones among treatments (anti–PD-1, VEGFRi, anti–PD-1 + VEGFRi, and anti-CD3/CD28/CD2) in CD8^+^ (i) and CD4^+^ (ii) T cells. **E,** Scatter plot showing the expansion levels of T cell clones in control and different treatments. Clonal expansion or contraction is measured as the ratio of cells from a clonotype to the number of T cells. Each point represents a T cell clone from an individual patient. For each clone, Fisher exact test was performed to evaluate the statistical significance of expansion or contraction, and clones with FDR ≤0.05 were considered significantly altered and labeled in the figure. **F,** Gene set enrichment analysis (GSEA) of pathways upregulated or downregulated by the treatments in selected CD8^+^ and CD4^+^ clones. Heatmap shows the GSEA results at the clone level for the five largest clones of each patient (RCC_4–RCC_8). The most prevalent cell phenotype (cluster) in the control condition is indicated for each clone. Signatures with FDR ≤0.01 are highlighted within the heatmap tiles. **G,** A dot plot showing the proportion of cell at exhausted and activated cluster in control and each treatment condition for the largest clones, labeling those clones that are less responsive to anti-CD3/CD28/CD2. The clones are shown in the same order as in **F**. **H,** A volcano plot showing the results of Fisher exact test, identifying upregulated genes (FDR ≤0.05; log_2_FC ≥ 0.25; Wilcoxon rank-sum test) that are overrepresented in anti-CD3/CD28/CD2-responsive clones compared with less responsive clones. Categorization to “responsive” and “less responsive” is based on the analysis presented in **G**. FC, fold change.

Expanded clonotypes (hyperexpanded clones, defined as clones with frequencies between 0.1 and 1, and large clones, defined as clones with frequencies between 0.01 and 0.1) reflect T cell clonal expansion, often linked to antigen-driven immune responses and potential tumor reactivity. We predominantly found expanded clonotypes in the exhausted cluster but also within the TEMRA clusters of CD8^+^ cells, as well as in the cytotoxic and exhausted clusters of CD4^+^ cells (Fig. 6A and B; Supplementary Fig. S6A and S6B). Notably, these clonotypes were largely confined to specific phenotypic states in the untreated control condition, indicating a restriction to single clusters rather than dispersion across multiple cell states (Supplementary Fig. S6C). CD8^+^ T cells exhibited a higher relative abundance of expanded clones than CD4^+^ T cells, encompassing both hyperexpanded and large clonotypes, whereas CD4^+^ T cells contained only large clones.

We further examined the relative clonal abundance and identified hyperexpanded clones in three patients (RCC_4, RCC_5, and RCC_6), comprising approximately 15% to 25% of the total CD8^+^ TCR repertoire (Fig. 6B). Across all patients, the hyperexpanded and large clones collectively accounted for roughly 50% of the CD8^+^ TCR repertoire. In contrast, most patients displayed the majority of their CD4^+^ TCR clones in a small size category, with only about 10% to 15% classified as medium-sized on average. However, patients RCC_1 and RCC_7 showed significant clonal expansion in their CD4^+^ TCR repertoire, with large clones making up approximately 15% and 20%, respectively. The expanded clones in patient RCC_1 predominantly showed an exhausted phenotype, whereas in patient RCC_7, they mainly exhibited a cytotoxic phenotype (Supplementary Fig. S6A).

Consistent with the high intratumoral clonal expansion, TCR clonality was higher in the exhausted, TEMRA, Tem/rm, and Tem clusters of CD8^+^ T cells (Fig. 6C; Supplementary Fig. S6A), which reflects prior antigen experience. Similarly, higher clonality was observed in the exhausted cytotoxic and cytotoxic clusters of CD4^+^ T cells. In CD8^+^ T cells, cycling cells included expanded clonotypes shared with exhausted cells, indicating that proliferation within exhausted cells is not entirely suppressed, as reported previously (15).

Our analysis of how treatment responses affected clonotype frequency revealed small but noticeable changes (Fig. 6D). Further analysis at the single clonotype level showed that under VEGFRi and combination treatments, seven and 12 CD8^+^ clonotypes, respectively, contracted significantly, with most displaying an exhausted phenotype (Fig. 6E). Additionally, when considering the results that were not statistically significant, exhausted CD8^+^ clones consistently contracted in response to VEGFRi and combination treatments, a pattern not observed following anti–PD-1 or anti-CD3/CD28/CD2 treatment.

As expanded CD8^+^ T cells represent potential tumor-targeting specificity, we further explored their transcriptional response to various treatments through pathway analysis of the five most expanded clonotypes from five individuals (25 clones in total; Fig. 6F). These clones exhibited diverse cell states in the control condition, including exhausted, TEMRA, Tem, and Tem/rm CD8^+^ as well as cytotoxic CD4^+^ T cells. Under anti–PD-1 treatment, the INF-γ response pathway was downregulated in 2 of 25 clones, with minimal or no response observed in the remaining clones. In contrast, VEGFRi and anti–PD-1 + VEGFRi treatments induced downregulation of the IFN response or inflammation pathways in most clones, as well as downregulation of the IL6–JAK–STAT3 and IL2–STAT5 signaling pathways in a subset of clones. Among the analyzed pathways, the MYC targets pathway was the only one upregulated in response to VEGFRi treatment. Anti-CD3/CD28/CD2 upregulated all 10 pathways, with notable variations observed among the 25 largest clones.

We further investigated these responses, noting a strong activation in both CD4^+^ and CD8^+^ T cells, as evident by the formation of a new cluster in UMAP (Fig. 4E and F). However, we observed variability in the extent to which individual T cell clones transitioned to the activated state (Fig. 6G), with the exhausted CD8^+^ clones and 3_TEMRA clone from patient RCC_6 showing resistance to activation. Based on their transition extent, we classified the 25 clones into “less responsive” and “anti-CD3/CD28/CD2–responsive” groups (Fig. 6G). Comparison of the genes upregulated in response to anti-CD3/CD28/CD2 revealed that less responsive clones showed increased *IFITM1* expression, indicating a stronger IFN response, a pattern also observed in the clone-level pathway analysis. The upregulated genes unique to the responsive clones included markers of immune activation (*CTLA4*, *TNFRSF18*, *TNFRSF9*, *TNFRSF4*, *IL2RA*, *CD83*, and *CCL3*), inflammation (*NFKB1*, *NFKB2*, *RELB*, *LTA*, and *ID2*), and metabolic reprogramming (*GAPDH*, *PKM*, *RPLPO*, *HSP90AB1*, *HSPB1*, and *IMPDH2*; Fig. 6H). These results suggest that although not activated by anti–PD-1 or VEGFRi, some of the largest tumor-infiltrating T cell clones retain the capacity to respond to strong TCR stimulation (anti-CD3/CD28/CD2), indicating their ability to respond to TCR triggering in the TME (35). Notably, this response depends on the clone phenotype, with exhausted, potentially tumor-reactive clones displaying weaker activation potential. The reduced activation and heightened IFN response suggests that exhausted CD8^+^ clones may respond to IFN-γ released by activated cells but remain unable to be activated via the TCR pathway.

### Immune cell communication within the TME

We aimed to further understand attenuated T cell activation in response to clinically relevant treatments and hypothesized that both the intrinsic features of T cells and cell–cell communication, via direct cellular interactions and secreted factors, may contribute to resistance. Cytokine profiling revealed that RCC TME exhibited high levels of multiple cytokines, chemokines, and interleukins (e.g., MIF, CCL2/3/4/5, CXCL10/12, IFN-γ, TNF, and IL15), in the absence of the treatments, reflecting abundant immune infiltration and baseline activation (Supplementary Fig. S4A). Conversely, the RCC TME was also enriched with immunomodulatory and angiogenic factors such as IL8 (*CXCL8*) and VEGF (36, 37).

**Figure 7.**
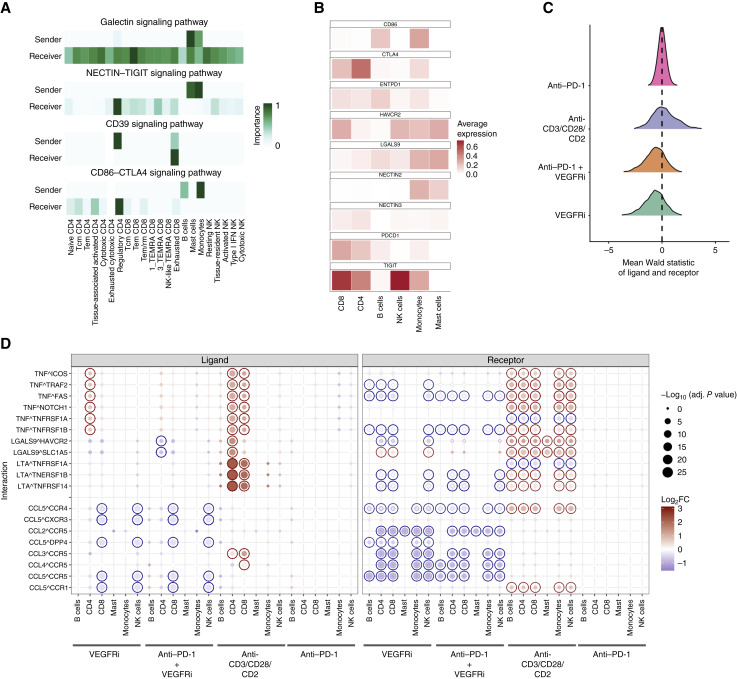
Cell–cell communication in the RCC TME. **A,** Heatmap showing cell–cell communication network for selected signaling pathways in the untreated, control condition. Normalized pathway network centrality scores (importance) are visualized for galectin, NECTIN-TIGIT, CD39, and CD86–CTLA4 signaling pathways, showing signal for cell types as “sender” and “receiver.” More intense color indicates higher relative “sender” or “receiver” signal. **B,** Heatmap showing the average expression of selected ligands and receptors in the control for specified immune cell types. **C,** Ridge plot showing the pseudobulk analysis comparing each treatment with the control condition. The change in the ligand–receptor expression is expressed as mean Wald statistic, in which positive values denote increase and negative values decrease in ligand–receptor expression. **D,** The results of the pseudobulk analysis comparing each treatment with the control condition were visualized as a dot plot. In this plot, each row represents a ligand–receptor pair, and each column corresponds to a cell type and treatment combination. Ligand and receptor results were visualized in separate panels. Dot color indicates the log_2_ FC between treatment and control, whereas dot size reflects −log_10_ FDR. Dots with FDR ≤0.25 were highlighted with a circle, in which circle color denotes the direction of change: red for higher expression in treatment and blue for lower expression. FC, fold change.

We first investigated cell–cell communication from single-cell data in detail using CellChat (38) and inferred putative interactions between different T and NK cell populations, myeloid cells, and B cells in the control condition. We identified ubiquitous inflammatory cytokine signaling mediated by MIF, CCL, and CXCL pathways and immunomodulatory signaling via TGF-β and galectin pathways (Fig. 7A; Supplementary Fig. S7A; Supplementary Tables S16 and S17). Predicted inhibitory interactions among T cell subsets, monocytes, and mast cells additionally occurred via the NECTIN–TIGIT, CD39 (*ENTPD1*), and CD86–CD28/CTLA4 pathways (Fig. 7A and B; Supplementary Fig. S7B). Monocytes and mast cells were inferred to secrete Galectin-9C (*LGALS9*; Fig. 7A and B).

We then examined putatively deregulated ligand–receptor pairs in response to the treatments across CD4^+^ and CD8^+^ T, NK, B, and myeloid cell populations using LIANA+ (23, 24). Anti-CD3/CD28/CD2 induced a strong increase in numerous ligands and receptors, whereas the opposite was evident for VEGFRi and combination treatment, and anti–PD-1 response was negligible (Fig. 7C). The most significantly upregulated interactions following anti-CD3/CD28/CD2 included TNF family interactions and galectin-mediated (*LGALS1/9*) inhibitory interactions (Fig. 7D; Supplementary Fig. S7C; Supplementary Tables S18–S21), along with increase in IFN-γ, chemokines (*CCL3/4*), and their receptors (Supplementary Fig. S7C; Supplementary Tables S18–S21). Checkpoint inhibitory molecules (*LAG3*, *CTLA4*, and *HAVCR2*) were mainly induced in T cells and NK cells (Supplementary Fig. S7C; Supplementary Tables S18–S21). VEGFRi alone, and in combination with anti–PD-1, downregulated expression of chemokines and their receptors, primarily driven by reduced *CCL5* expression in CD8^+^ and NK cells (Fig. 7D; Supplementary Fig. S7D and S7E; Supplementary Tables S18–S21), reflecting treatment-induced downregulation of inflammatory response.

## Discussion

RCC presents a unique challenge in tumor immunology due to its paradoxical combination of low mutational burden yet high immune infiltration and cytolytic activity compared with other tumor types (9, 10, 39). Despite presenting features of an immunoreactive TME, high immune infiltration and related inflammation may correlate with poor prognosis and outcome following nephrectomy (11). Our study explores the interplay between TME modulation and immune cell dynamics under treatments combining immune checkpoint inhibitors and VEGFRis.

The RCC TME exhibited characteristics of both immune-activated and suppressed cytokine milieus, with abundant infiltration of markedly heterogeneous CD8^+^ and CD4^+^ T cell populations. Clonally expanded T cells were mainly observed in exhausted, TEMRA, Tem, and cytotoxic cell states, suggesting preexisting tumor immunity and ongoing intratumoral T cell responses. Further clonotype analysis revealed that T cells were predominantly restricted to specific cell phenotypes, rather than being dispersed across different states. Exhausted CD8^+^ T cells exhibited features of dysfunction and terminal exhaustion (12, 25, 28), despite the early local tumor stage (stages I–III). In our model, these cells engaged in inhibitory interactions with regulatory CD4^+^ T cells, monocytes, and mast cells, consistent with previously described observations involving tumor-associated macrophages and renal tumor cells in advanced RCC (14, 16).

Patient-derived *ex vivo* culture systems have emerged to model treatment responses to immunotherapies (18, 35, 40–42). These models maintain patient-specific TME features while enabling the functional assessment of treatments and baseline TME characteristics. To address gaps in our understanding of treatment responses in RCC, we developed an *ex vivo* culture platform using patient-derived RCC tissue to study early immunologic responses in the TME. We first investigated the introduction of a potent T cell activator, ImmunoCult (anti-CD3/CD28/CD2), into the RCC TME. This treatment resulted in substantial alterations in the immune landscape, characterized by the emergence of activated T cell subsets. These subsets displayed markers of robust activation, including upregulation of key immune signaling pathways, such as IFN-γ, TNF-α, and IL2. Notably, both CD8^+^ and CD4^+^ T cells exhibited phenotypic changes that were indicative of heightened immune activity. These findings demonstrate that, despite the immunosuppressive cues and exhausted T cell presence in the TME, a strong immune activator can overcome these barriers and elicit a potent immune response. This activation was accompanied by an increase in the terminal exhaustion signature in T cells (16, 29), including elevated *PDCD1* (PD-1), *LAG3*, and *HAVCR2* (TIM-3) expression, alongside a reduction in the progenitor exhaustion signature. Notably, intratumoral T cells with a progenitor or stem-like state are suggested to differentiate toward terminally exhausted progeny during checkpoint inhibitor therapy and are associated with better clinical outcomes (29, 43, 44). We found that T cells responded differently to activation stimuli in the TME depending on their phenotypic state, with exhausted CD8^+^ T cells being particularly resistant to the activation switch. These results indicate that the state of the tumor-reactive CD8^+^ T cells, rather than the mere abundance of infiltrating lymphocytes, may be important for immunotherapy responses. Furthermore, exhausted CD8^+^ T cells expressed several checkpoint molecules, including PD-1, LAG-3, and TIM-3, indicating profound ongoing immune suppression. We observed limited immune activation following anti–PD-1 checkpoint inhibitor treatment in the TME, suggesting the need for combination therapies targeting multiple immune checkpoints to achieve effective immune cell reinvigoration in RCC.

Antiangiogenic and immune checkpoint therapies are the standard-of-care for patients with first-line metastatic ccRCC. The rationale for their synergistic combination stems from evidence that VEGF, in addition to causing aberrant vasculature, modulates both adaptive and innate immune responses and contributes to immunosuppression (45). VEGF facilitates the recruitment of immunosuppressive cell populations and inhibits adaptive immune cell trafficking to the tumor. Moreover, VEGF directly inhibits T cell proliferation, activation, and effector functions in the TME (46–49). Our *ex vivo* patient-derived tumor model partially recapitulated these characteristics, including the immunosuppressive cytokine milieu and heterogeneity of immune cells, comprising suppressive cell types and exhausted T cell states. We believe that our model also preserves other main cell types of the renal cancer tissue, such as tumor and endothelial cells. Although these cell types were not directly analyzed at the single-cell level, they may have contributed to detected immunologic responses. We observed that VEGF receptor inhibition led to an unexpectedly significant decrease in several cytokines, accompanied by a concurrent downregulation of immune signaling activities in T cells within the TME. Because infiltrating immune cells largely did not express VEGF receptors in our model, they were unlikely to be directly modulated by the treatment. Therefore, this response is likely driven primarily by nonimmune cell types, such as endothelial cells (50, 51). These findings highlight the intricate balance of cytokine signaling within the TME and its impact on immune cell function. We also observed that VEGFRi reduced clone size, mediating CD8^+^ T cell clonal contraction, and demonstrated that its combination with anti–PD-1 achieves a comparable effect. Clinical studies have shown that VEGF inhibitors, while facilitating immune cell infiltration, may primarily function by normalizing tumor vasculature rather than directly enhancing TME-specific immune activation (52). Such normalization may support T cell trafficking and survival during transit.

Despite careful planning, our study has several limitations. Although our platform preserves the immune contexture of the TME, it does not maintain its native architecture, potentially obscuring insights into immune cell–tumor interactions. In addition, tissue dissociation may lead to the loss of critical structures such as tertiary lymphoid formations, which are known to contribute to immune activation (53, 54). Treatment responses may also have been influenced by the ECM and associated growth factors used in the culture system. Even though we identified T cell states suggestive of cytotoxic potential or intratumoral tumor-targeting activity, we do not provide experimental validation for direct tumor killing. Future research should focus on *in vivo* models to directly link immune activation with tumor killing and investigate the differential effects of VEGF inhibitors on immune cell trafficking and functionality.

In conclusion, our results highlight the multifaceted nature of RCC immunotherapy, in which vascular normalization, immune cell activation, and suppression of inhibitory pathways intersect. These findings emphasize the need for tailored combination strategies to overcome TME-mediated immune evasion and pave the way for future investigations into checkpoint inhibitor combinations, immune cell dynamics, and TME-specific mechanisms in RCC.

## Supplementary Material

Supplementary Fig. S1 Patient-derived ex vivo model and main immune cell subsets per patient (related to Fig. 1).

Supplementary Fig. S2 CD8+ T cell cluster annotation (related to Fig. 2).

Supplementary Fig. S3 CD4+ T cell and NK cell cluster annotation (Related to Fig. 3).

Supplementary Fig. S4 Patient-derived ex vivo model validation and anti-CD3/CD28/CD2 responses (related to Fig. 4).

Supplementary Fig. S5 Anti-PD1 (Pembrolizumab), VEGFR inhibitor (Axitinib) and their combination treatment responses in the RCC patient-derived ex vivo model (Related to Fig. 5)

Supplementary Fig. S6 Analysis of T cell clonotypes (related to Fig. 6)

Supplementary Fig.S7 Cell-cell communication in RCC TME (related to Fig 7.)

Supplementary Table S1 Cohort clinical characteristics

Supplementary Table S2 Supplementary Table S2

Supplementary Table S3 Top 50 differentially expressed genes (DEGs) for 14 CD8+ T cell clusters

Supplementary Table S4 Curated gene signatures used to characterize CD8+ T cells (Based on Bi et.al.)

Supplementary Table S5 Curated gene signatures used to characterize CD8+ T cells (Based on Sade-Feldman et.al.)

Supplementary Table S6 Top 50 differentially expressed genes (DEGs) for 14 CD4+ T cell clusters

Supplementary Table S7 Curated gene signatures used to characterize CD4+ T cells (Based on Bi et.al.)

Supplementary Table S8 Top 50 differentially expressed genes (DEGs) for 7 NK cell clusters

Supplementary Table S9 Effect size of the cytokines used in Cytokine panel per treatment

Supplementary Table S10 Differentially expressed genes (DEGs) in pseudobulked treatment vs control in CD8+ cells

Supplementary Table S11 Differentially expressed genes (DEGs) in pseudobulked treatment vs control in CD8+ cells

Supplementary Table S12 Differentially expressed genes (DEGs) in pseudobulked treatment vs control in CD4+ cells

Supplementary Table S13 Enriched pathways in differentially expressed genes (DEGs) in treatment vs control in CD4+ cells according to Metascape

Supplementary Table S14 Differentially expressed genes (DEGs) in pseudobulked treatment vs control in NK cells, B cells and Myeloid population

Supplementary Table S15 Enriched pathways in differentially expressed genes (DEGs) in treatment vs control in B, myloid and NK cells according to Metascape

Supplementary Table S16 Secreted Signaling interactions according to Cell-chat

Supplementary Table S17 Cell-cell contact according to Cell-chat

Supplementary Table S18 Cell-cell interaction changes in anti-PD1 vs Control according to Liana+

Supplementary Table S19 Cell-cell interaction changes in VEGFRi vs Control according to Liana+

Supplementary Table S20 Cell-cell interaction changes in anti-PD1 + VEGFRi vs Control according to Liana+

Supplementary Table S21 Cell-cell interaction changes in anti-CD3/CD28/CD2 vs Control according to Liana+

## Data Availability

All data needed to evaluate the conclusions in the article are present in the paper and/or Supplementary Materials. The feature-barcode matrices, Seurat objects generated from the scRNA-seq in this study, and R scripts and code used for data analysis are available at Zenodo (accession 10.5281/zenodo.15388467). Sensitive scRNA-seq data (FASTQ files) generated in this study are available at FEGA (https://research.csc.fi/service/fega/) under accession number EGAD50000001934 (https://sd-apply.csc.fi/catalogue).
