# Supplementary material for: Functionally Characterizing the Renal Cell Carcinoma Tumor-Immune Microenvironment via Patient-Derived Ex Vivo Models
Source: Cancer Res Commun. 2026 Feb 26;6(2):402–20. doi: 10.1158/2767-9764.CRC-25-0447 (PMC13138221; doi:10.1158/2767-9764.CRC-25-0447)
Supplement: Supplementary Fig. S1 — Patient-derived ex vivo model and main immune cell subsets per patient (related to Fig. 1). [file crc-25-0447_supplementary_fig.s1_suppsf1.pdf]

A

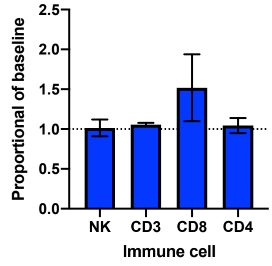

B

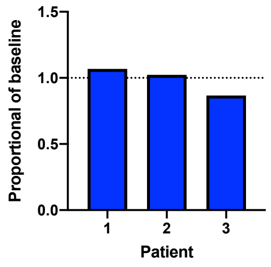

C

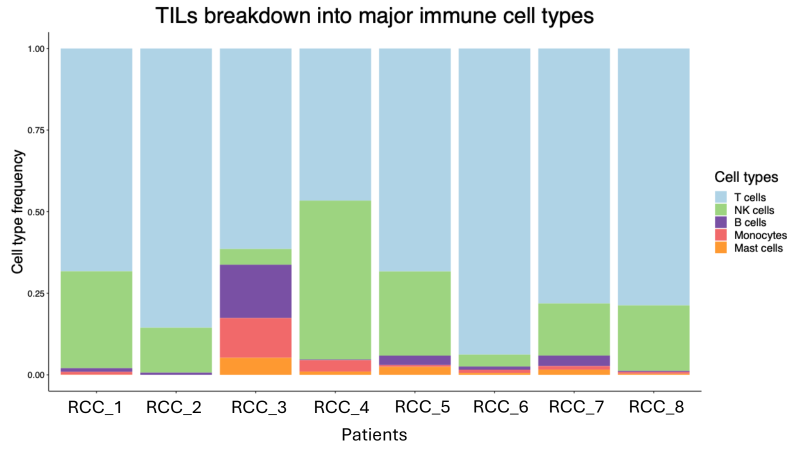

D

i

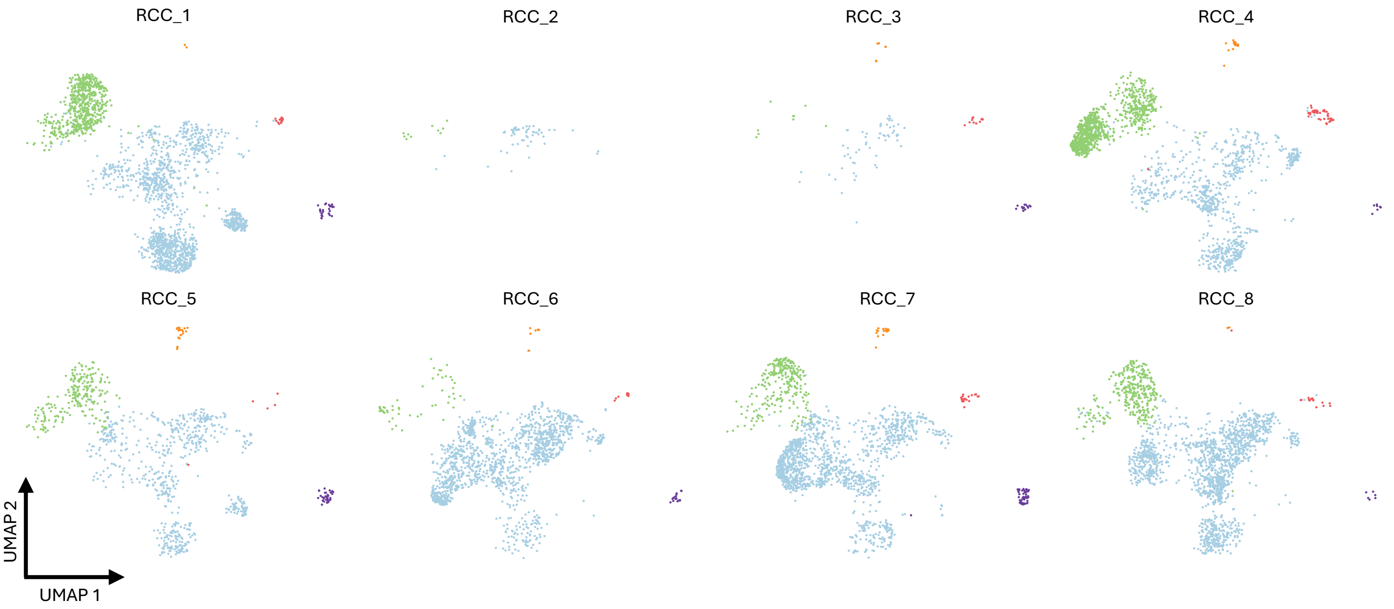

ii

	T cells	NK cells	B cells	Monocytes	Mast cells	Total number of cells
RCC_1	1417	616	28	18	2	2081
RCC_2	56	10	0	0	0	66
RCC_3	59	9	14	11	6	99
RCC_4	825	925	7	50	15	1822
RCC_5	617	225	35	5	21	903
RCC_6	1360	54	16	14	7	1451
RCC_7	1355	264	50	20	18	1707
RCC_8	1513	338	6	15	2	1874

E

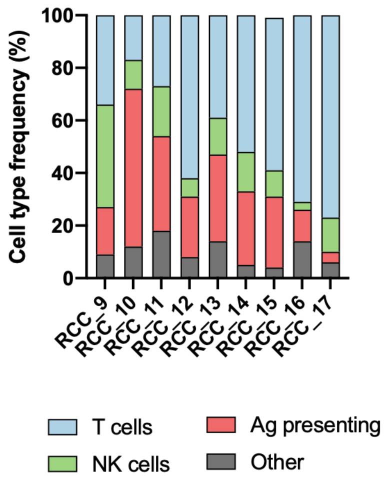

Supplementary Fig. S1

F

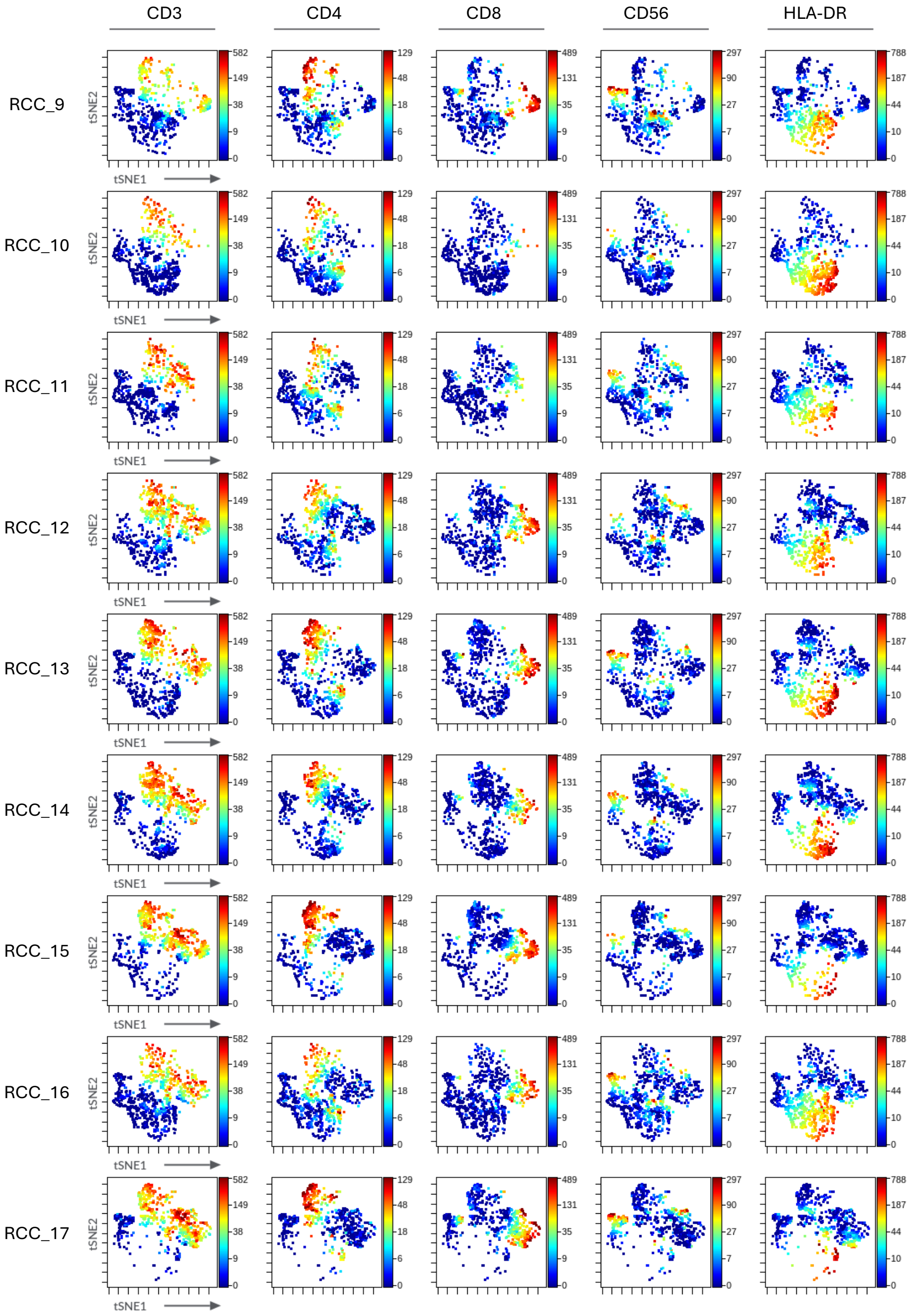
CD3
CD4
CD8
CD56
HLA-DR
RCC_9
RCC_10
RCC_11
RCC_12
RCC_13
RCC_14
RCC_15
RCC_16
RCC_17
tSNE1
tSNE2
582 149 38 9 0
129 48 18 6 0
489 131 35 9 0
297 90 27 7 0
788 187 44 10 0

G

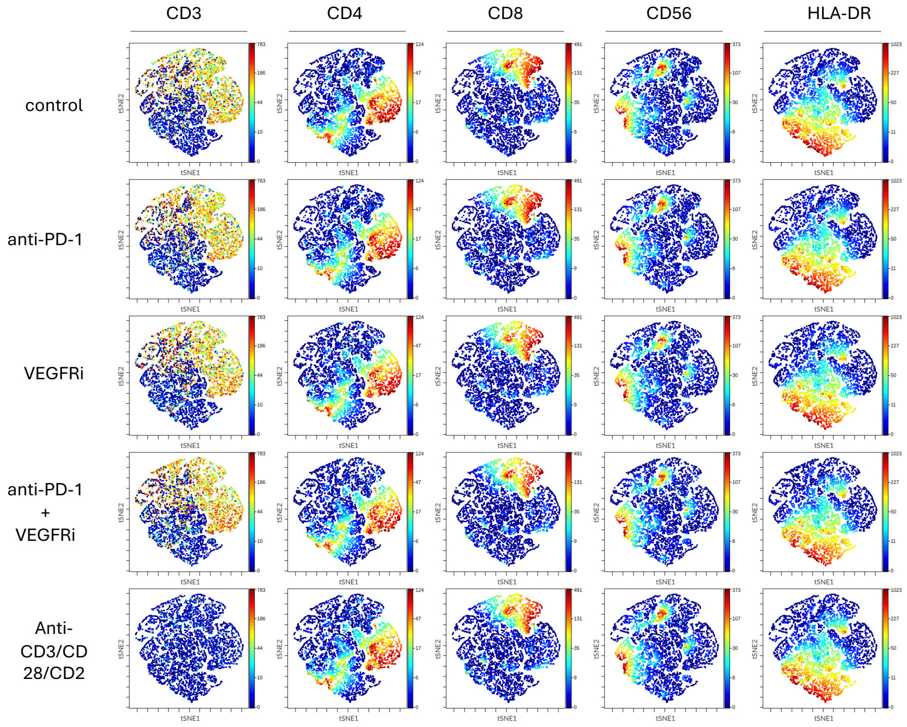

Supplementary Fig 1. Patient-derived *ex vivo* model and main immune cell subsets per patient (related to Fig. 1). (A) Patient-derived *ex vivo* model retains immune cell contexture for the duration of the treatment. Proportion of T cell and NK cell immune cells following incubation in comparison to fresh tumor dissociate, n= 4. (B) Patient-derived *ex vivo* model retains immune cell viability for the duration of the treatment. Proportion of immune cell viability following incubation in comparison to fresh tumor dissociate, n= 3. Immune cell contexture and viability was measured with flow cytometry, using AnnexinV and 7-AAD as viability markers. (C) Bar plot highlighting the immune cell types heterogeneity between the 8 patients in scRNA-seq cohort (RCC_1 - RCC_8). (D) UMAP displaying the heterogeneity between the 8 patients (RCC_1 - RCC_8) in scRNA-seq cohort in the control condition (i), and table (ii) highlighting the total number of cells each patient has in the control condition, together with number of cells per each cluster. (E) Relative immune cell type frequency in patients RCC_9 - RCC_17 as measured using CyTOF. (F) tSNE plots of patients RCC_9 - RCC_17 CD45+ cells at control. (G) tSNE plots of patients RCC_9 - RCC_17 CD45+ cells at control and following anti-PD1, VEGFRi, anti-PD1 + VEGFRi combination and anti-CD3/CD28/CD2 treatments.
