# Supplementary material for: Functionally Characterizing the Renal Cell Carcinoma Tumor-Immune Microenvironment via Patient-Derived Ex Vivo Models
Source: Cancer Res Commun. 2026 Feb 26;6(2):402–20. doi: 10.1158/2767-9764.CRC-25-0447 (PMC13138221; doi:10.1158/2767-9764.CRC-25-0447)
Supplement: Supplementary Fig. S2 — CD8+ T cell cluster annotation (related to Fig. 2). [file crc-25-0447_supplementary_fig.s2_suppsf2.pdf]

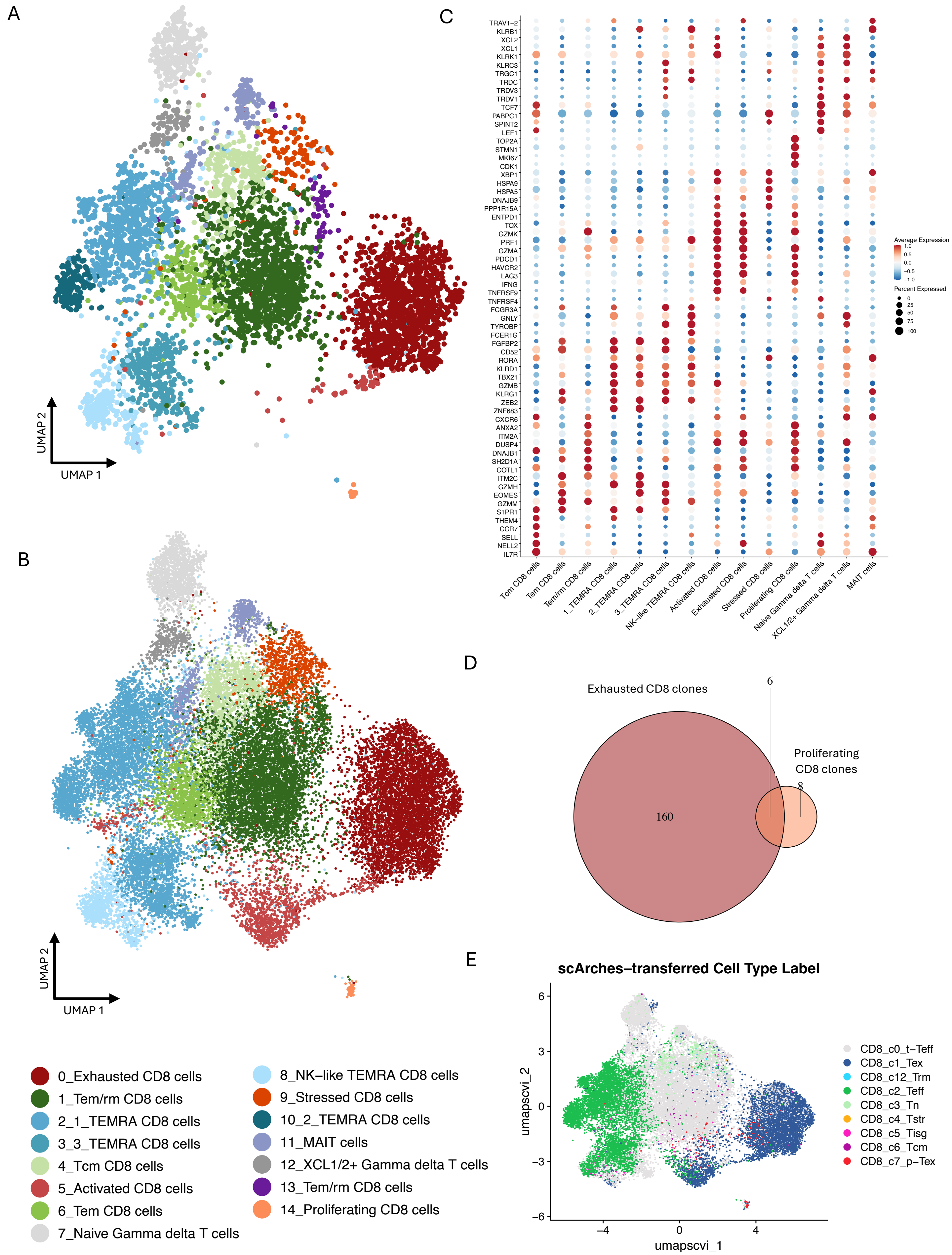

Supplementary Fig. S2

Supplementary Fig. S2. CD8+ T cell cluster annotation (related to Fig. 2). Uniform manifold approximation and projection (UMAP) of CD8+ T cells captured across eight patient samples (RCC_1 – RCC_8) baseline (control, 5177 CD8+ T cells) (A), and in all conditions (control, anti-PD-1, VEGFRi, anti-PD-1 + VEGFRi and anti-CD3/CD28/CD2) (B), colored by annotated cell types, including separate TEMRA sub-clusters. (C) Dot plot displaying expression of selected markers used in annotating across CD8+ T cell clusters. (D) Plot showing the shared clones between proliferating and exhausted CD8+ T cell clusters, highlighting that 43% of clones found in the proliferating cluster can also be identified in the exhausted one. (E) UMAP showing CD8+ T cells identified in the study plotted onto the PanAtlas data, confirming our cell type annotation.
