# Supplementary material for: Functionally Characterizing the Renal Cell Carcinoma Tumor-Immune Microenvironment via Patient-Derived Ex Vivo Models
Source: Cancer Res Commun. 2026 Feb 26;6(2):402–20. doi: 10.1158/2767-9764.CRC-25-0447 (PMC13138221; doi:10.1158/2767-9764.CRC-25-0447)
Supplement: Supplementary Fig. S3 — CD4+ T cell and NK cell cluster annotation (Related to Fig. 3). [file crc-25-0447_supplementary_fig.s3_suppsf3.pdf]

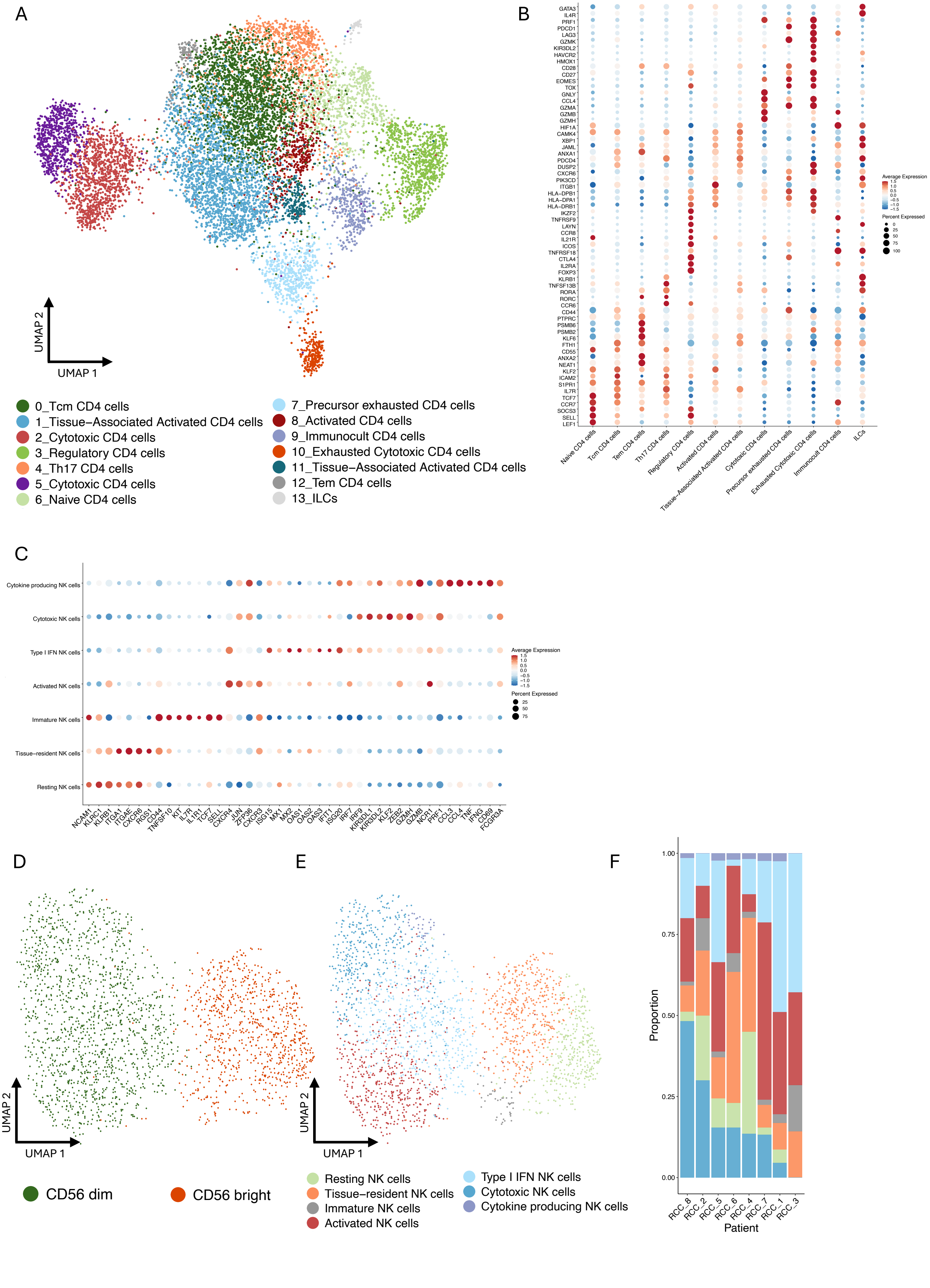

Supplementary Fig. S3

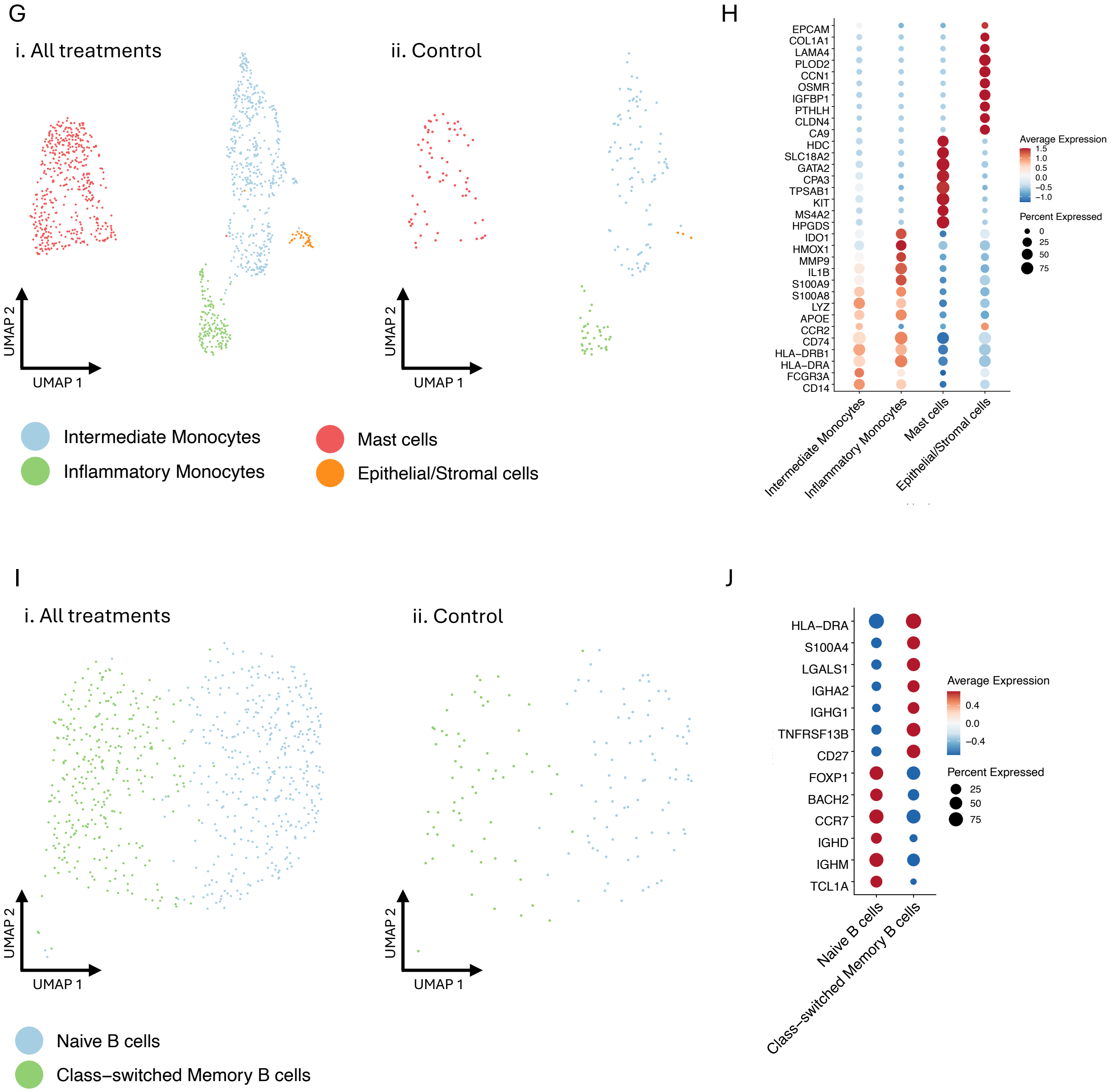

Supplementary Fig. S3. CD4+ T cell and NK cell cluster annotation (Related to Fig. 3). (A) Uniform manifold approximation and projection (UMAP) of CD4+ T cells captured across eight patient samples (RCC_1 – RCC_8), and 5 treatment conditions (control, anti-PD1, VEGFRi, anti-PD1 + VEGFRi and anti-CD3/CD28/CD2) colored by annotated cell types. (B) Dot plot displaying expression of selected markers used in naming across CD4+ T cell clusters. (C) Dot plot displaying expression of selected markers used in annotating across NK cell clusters. (D) UMAP of NK cells captured across eight patient samples (RCC_1 – RCC_8) in the control condition, displaying separation into CD56 dim and CD56 bright subsets. (E) UMAP of NK cells captured across eight patient samples (RCC_1 – RCC_8) in the control condition colored by characterized cell types. (F) Bar plot showing distribution of characterized NK cell types across all eight patients. (G) UMAP of myeloid cells captured across eight patient samples (RCC_1 – RCC_8) in all conditions (i.) and in control condition (ii.) colored by characterized cell types. (H) Dot plot displaying expression of selected markers used in annotating across myeloid clusters. (I) UMAP of B cells captured across eight patient samples (RCC_1 – RCC_8) in all conditions (i.) and in control condition (ii.) colored by characterized cell types. (J) Dot plot displaying expression of selected markers used in annotating across B cell clusters.
