# Supplementary material for: Functionally Characterizing the Renal Cell Carcinoma Tumor-Immune Microenvironment via Patient-Derived Ex Vivo Models
Source: Cancer Res Commun. 2026 Feb 26;6(2):402–20. doi: 10.1158/2767-9764.CRC-25-0447 (PMC13138221; doi:10.1158/2767-9764.CRC-25-0447)
Supplement: Supplementary Fig. S4 — Patient-derived ex vivo model validation and anti-CD3/CD28/CD2 responses (related to Fig. 4). [file crc-25-0447_supplementary_fig.s4_suppsf4.pdf]

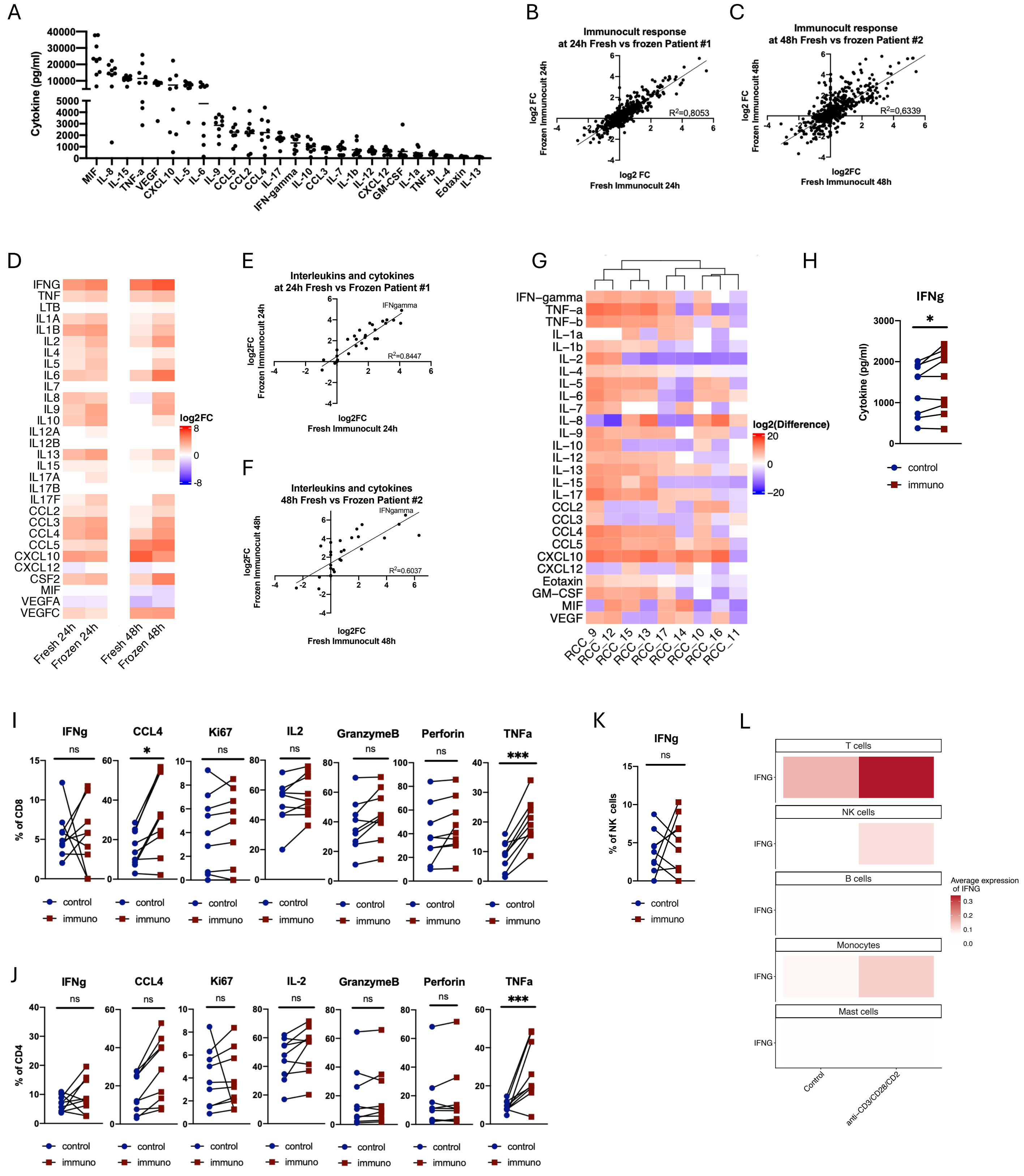

Supplementary Fig. S4

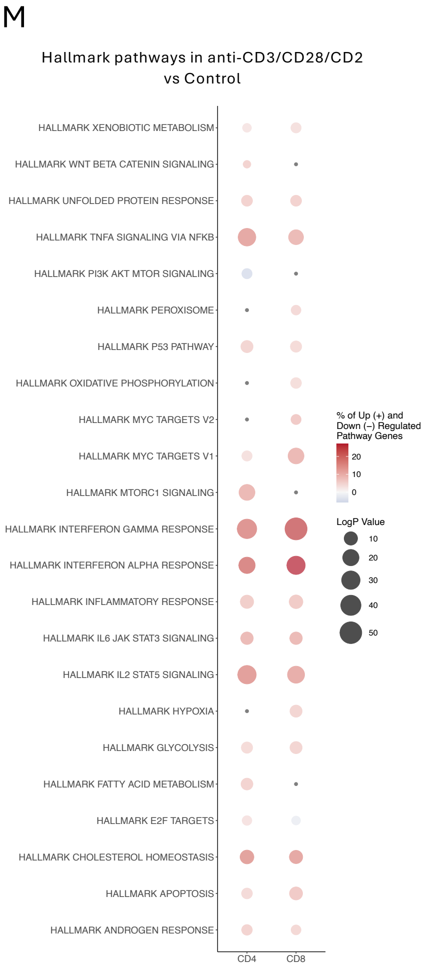
M
Hallmark pathways in anti-CD3/CD28/CD2
vs Control
HALLMARK XENOBIOTIC METABOLISM
HALLMARK WNT BETA CATENIN SIGNALING
HALLMARK UNFOLDED PROTEIN RESPONSE
HALLMARK TNFA SIGNALING VIA NFKB
HALLMARK PI3K AKT MTOR SIGNALING
HALLMARK PEROXISOME
HALLMARK P53 PATHWAY
HALLMARK OXIDATIVE PHOSPHORYLATION
HALLMARK MYC TARGETS V2
HALLMARK MYC TARGETS V1
HALLMARK MTORC1 SIGNALING
HALLMARK INTERFERON GAMMA RESPONSE
HALLMARK INTERFERON ALPHA RESPONSE
HALLMARK INFLAMMATORY RESPONSE
HALLMARK IL6 JAK STAT3 SIGNALING
HALLMARK IL2 STAT5 SIGNALING
HALLMARK HYPOXIA
HALLMARK GLYCOLYSIS
HALLMARK FATTY ACID METABOLISM
HALLMARK E2F TARGETS
HALLMARK CHOLESTEROL HOMEOSTASIS
HALLMARK APOPTOSIS
HALLMARK ANDROGEN RESPONSE
CD4
CD8
% of Up (+) and Down (–) Regulated Pathway Genes
20
10
0
LogP Value
10
20
30
40
50

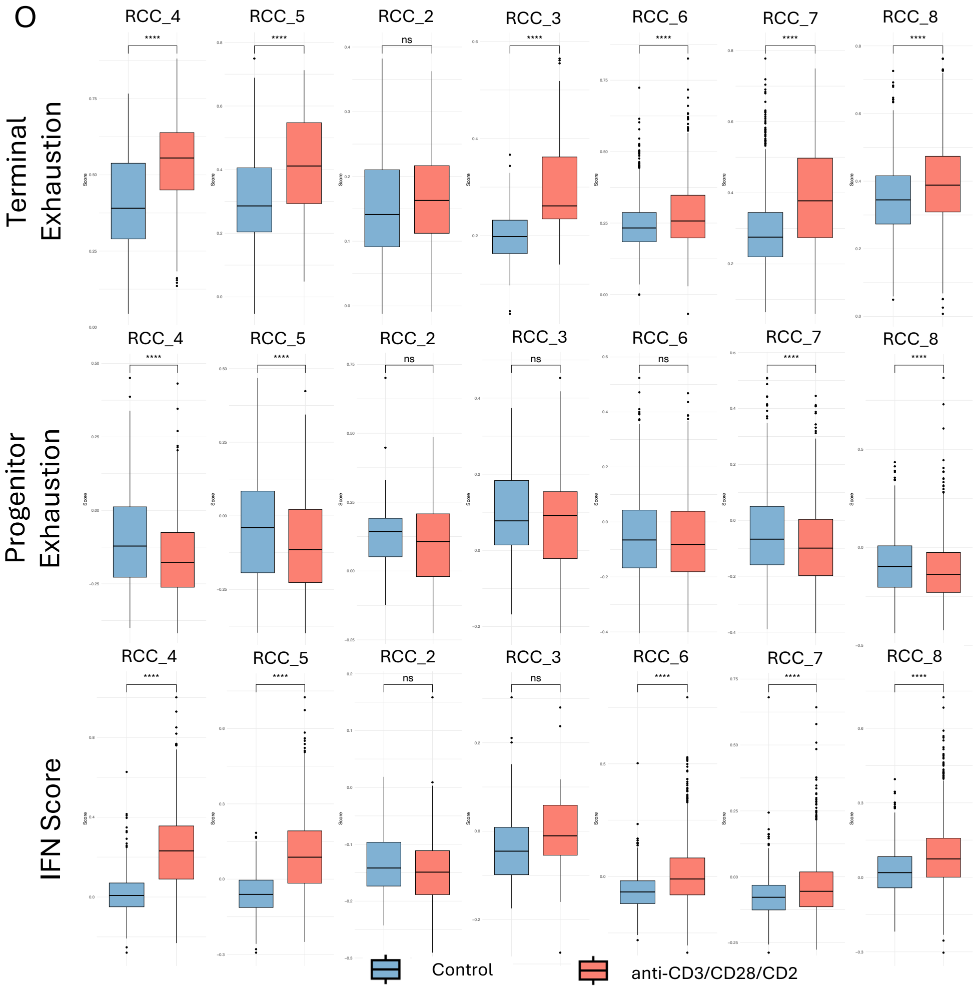
O
RCC_4
RCC_5
RCC_2
RCC_3
RCC_6
RCC_7
RCC_8
Terminal Exhaustion
Progenitor Exhaustion
IFN Score
****
ns
Control
anti-CD3/CD28/CD2

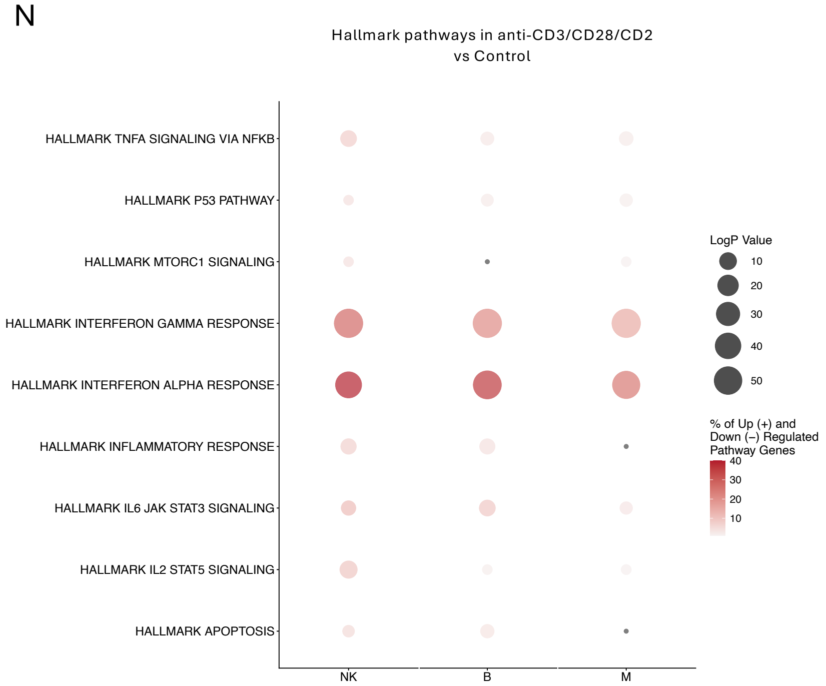
N
Hallmark pathways in anti-CD3/CD28/CD2
vs Control
HALLMARK TNFA SIGNALING VIA NFKB
HALLMARK P53 PATHWAY
HALLMARK MTORC1 SIGNALING
HALLMARK INTERFERON GAMMA RESPONSE
HALLMARK INTERFERON ALPHA RESPONSE
HALLMARK INFLAMMATORY RESPONSE
HALLMARK IL6 JAK STAT3 SIGNALING
HALLMARK IL2 STAT5 SIGNALING
HALLMARK APOPTOSIS
NK
B
M
LogP Value
10
20
30
40
50
% of Up (+) and Down (–) Regulated Pathway Genes
40
30
20
10

Supplementary Fig. S4. Patient-derived *ex vivo* model validation and anti-CD3/CD28/CD2 responses (related to Fig. 4). (A) Immune cell functionality at control as analyzed by cytokine profiling. Analysis of patients RCC_9 - RCC_17 using customized BioRad 27plex cytokine and growth factor kit. Each dot represents an individual. (B, C) *Ex vivo* model validation showing immune cell functional activation in fresh sample in comparison to live-frozen sample following 24h (B) or 48h (C) treatment incubation. Immune cells were either untreated or activated using Immunocult (anti-CD3/CD28/CD2) human T cell activator and immune panel gene expression was measured using NanoSting platform. Scatter plots illustrate comparisons of treatment induced changes (log2 fold change) detected in fresh or frozen samples, where each dot represent regulation of a specified gene expression. (D, E, F) Immune cell functional activation in fresh samples and in live-frozen samples following 24h (D, E) or 48h (D, F) treatment incubation. Immunocult (anti-CD3/CD28/CD2) -mediated regulation in interleukins and cytokine, as measured using NanoString immune genes panel, is shown as heatmap (D) and scatter plot (E, F). (G) Heatmap displaying log2 converted difference (treatment - control) in cytokine concentration following anti-CD3/CD28/CD2 (Immunocult) treatment for each individual (RCC_9 - RCC_17) as measured using cytokine profiling. Patient responses are clustered using Wards clustering. (H) Cytokine profiling data for IFN-gamma of patients RCC_9 - RCC_17 at control and following anti-CD3/CD28/CD2 (Immunocult) treatment. (I-K) CyTOF analysis of anti-CD3/CD28/CD2 (Immunocult) treatment responses in CD8+ (I) and CD4+ (J) T cells and NK cells (K) for selected markers. Shown is percentage of marker positive cells in control and treatment condition. Each dot represents an individual (RCC_9 - RCC_17). Statistical significance was calculated using Graph Pad multiple t test, *ns* = not significant, * = $P < 0.05$, *** = $P < 0.005$. (L) Heatmap displaying the average expression of *IFNG* in control and anti-CD3/CD28/CD2 conditions, highlighting that the average expression of *IFNG* is higher in treated cells. (M, N) Dot plot of immunology-related hallmark pathways, comparing anti-CD3/CD28/CD2 (Immunocult) and control conditions in CD8+ and CD4+ T cells (M) or NK cells (NK), B cells (B) and myeloid cells (M) (N). Genes were identified using the FindMarkers method and included if the p-value was < 0.05. Circle size represents the LogP value, while color indicates the percentage of genes in our dataset that are upregulated (+) (in red). (O) Signature score distributions for terminally exhausted, progenitor exhausted and IFN signatures comparing control and anti-CD3/CD28/CD2 (Immunocult) in CD8+ T cells shown for each patient (RCC_2 - RCC_8). Significance of differential signature enrichment (p value) between subtypes was determined by two-sided Wilcoxon rank-sum test.
