# Supplementary material for: Functionally Characterizing the Renal Cell Carcinoma Tumor-Immune Microenvironment via Patient-Derived Ex Vivo Models
Source: Cancer Res Commun. 2026 Feb 26;6(2):402–20. doi: 10.1158/2767-9764.CRC-25-0447 (PMC13138221; doi:10.1158/2767-9764.CRC-25-0447)
Supplement: Supplementary Fig. S5 — Anti-PD1 (Pembrolizumab), VEGFR inhibitor (Axitinib) and their combination treatment responses in the RCC patient-derived ex vivo model (Related to Fig. 5) [file crc-25-0447_supplementary_fig.s5_suppsf5.pdf]

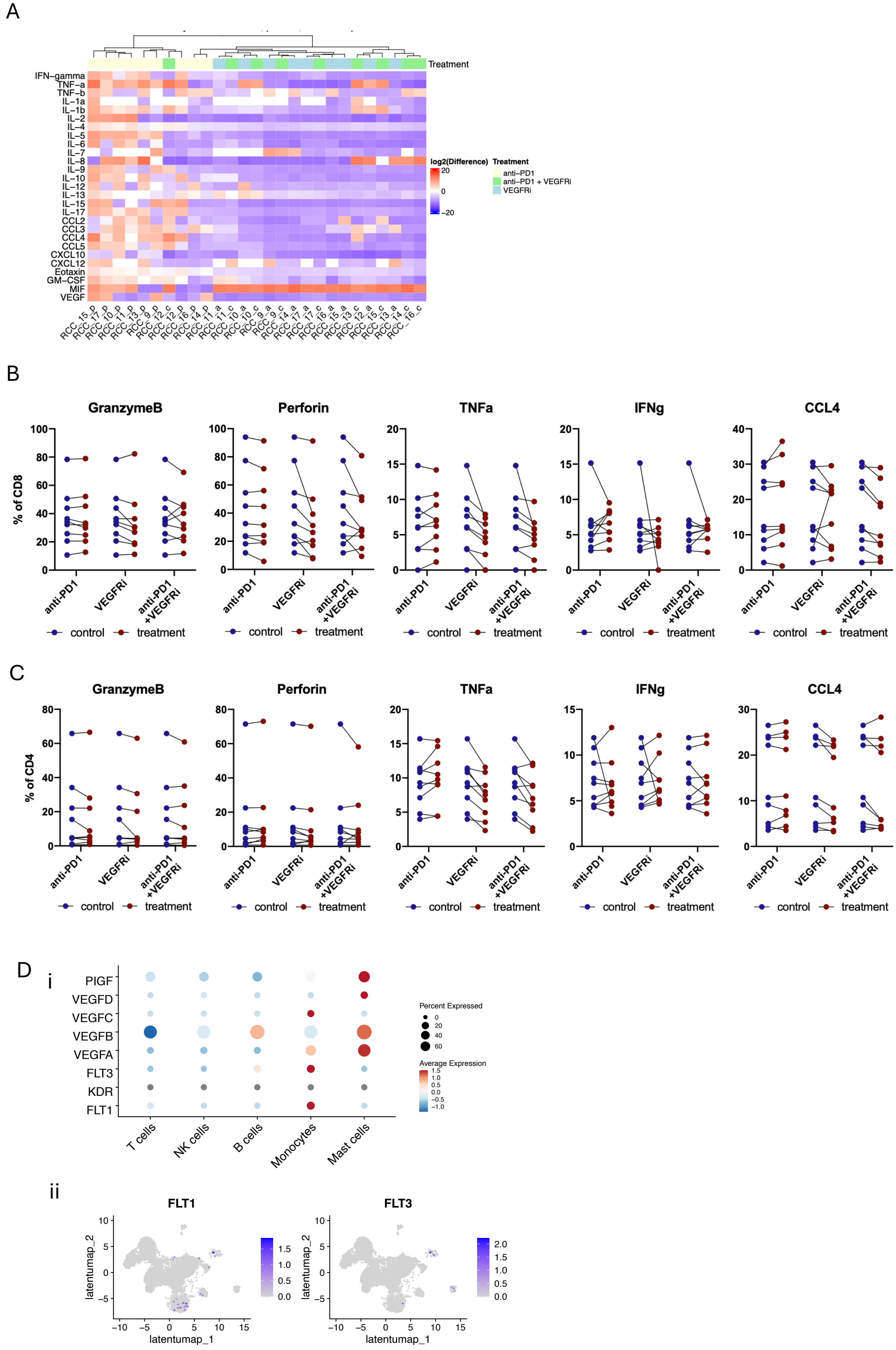

Supplementary Fig. S5

E

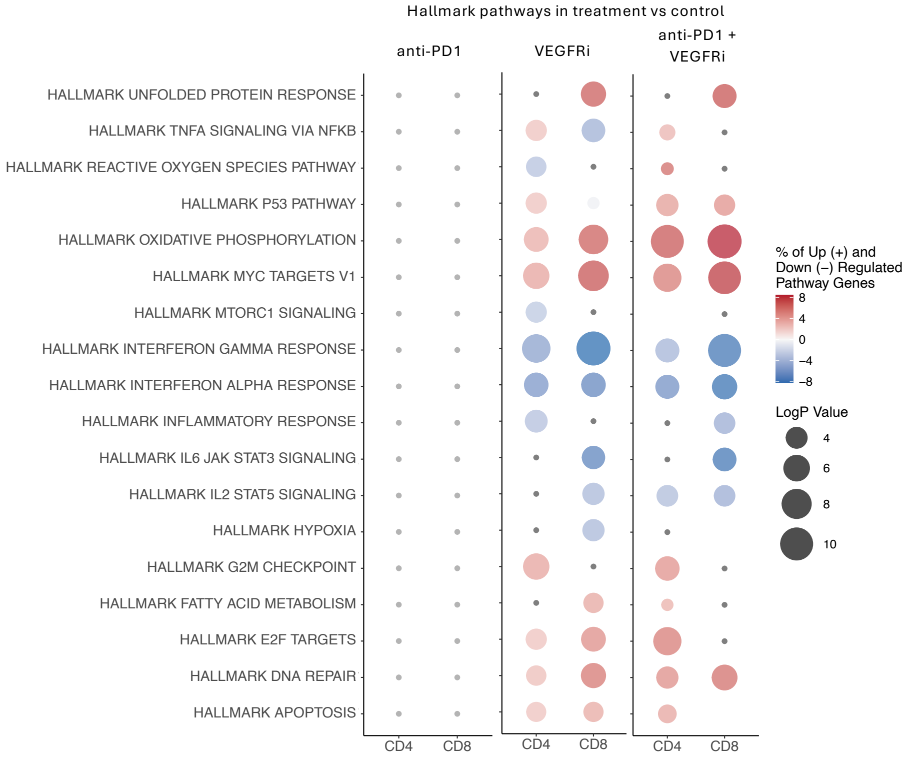
Hallmark pathways in treatment vs control
anti-PD1
VEGFRi
anti-PD1 + VEGFRi
HALLMARK UNFOLDED PROTEIN RESPONSE
HALLMARK TNFA SIGNALING VIA NFKB
HALLMARK REACTIVE OXYGEN SPECIES PATHWAY
HALLMARK P53 PATHWAY
HALLMARK OXIDATIVE PHOSPHORYLATION
HALLMARK MYC TARGETS V1
HALLMARK MTORC1 SIGNALING
HALLMARK INTERFERON GAMMA RESPONSE
HALLMARK INTERFERON ALPHA RESPONSE
HALLMARK INFLAMMATORY RESPONSE
HALLMARK IL6 JAK STAT3 SIGNALING
HALLMARK IL2 STAT5 SIGNALING
HALLMARK HYPOXIA
HALLMARK G2M CHECKPOINT
HALLMARK FATTY ACID METABOLISM
HALLMARK E2F TARGETS
HALLMARK DNA REPAIR
HALLMARK APOPTOSIS
CD4
CD8
% of Up (+) and Down (–) Regulated Pathway Genes
8
4
0
−4
−8
LogP Value
4
6
8
10

F

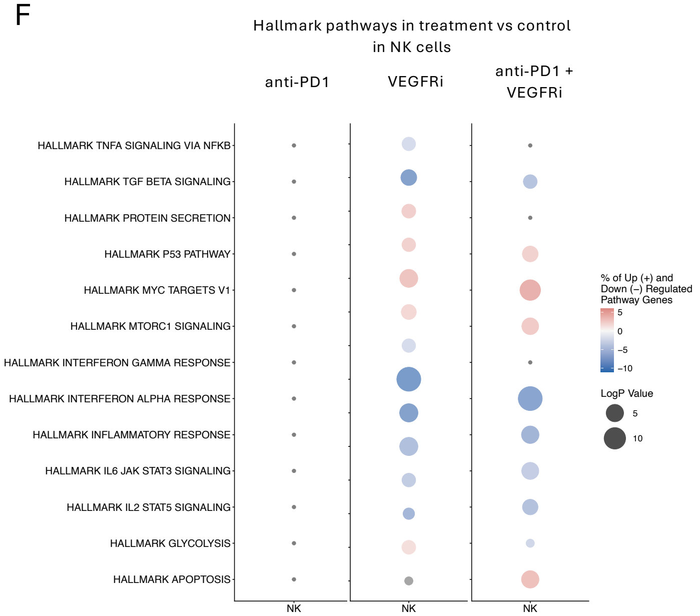
Hallmark pathways in treatment vs control in NK cells
anti-PD1
VEGFRi
anti-PD1 + VEGFRi
HALLMARK TNFA SIGNALING VIA NFKB
HALLMARK TGF BETA SIGNALING
HALLMARK PROTEIN SECRETION
HALLMARK P53 PATHWAY
HALLMARK MYC TARGETS V1
HALLMARK MTORC1 SIGNALING
HALLMARK INTERFERON GAMMA RESPONSE
HALLMARK INTERFERON ALPHA RESPONSE
HALLMARK INFLAMMATORY RESPONSE
HALLMARK IL6 JAK STAT3 SIGNALING
HALLMARK IL2 STAT5 SIGNALING
HALLMARK GLYCOLYSIS
HALLMARK APOPTOSIS
NK
% of Up (+) and Down (–) Regulated Pathway Genes
5
0
−5
−10
LogP Value
5
10

G

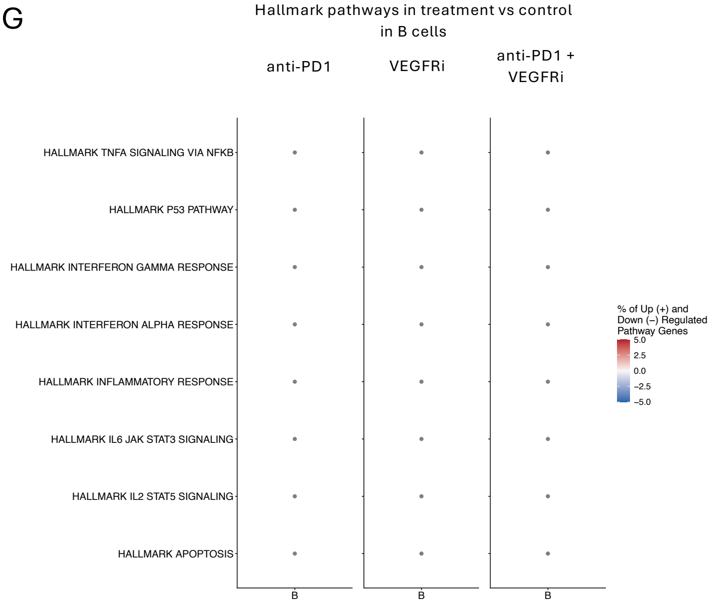
Hallmark pathways in treatment vs control in B cells
anti-PD1
VEGFRi
anti-PD1 + VEGFRi
HALLMARK TNFA SIGNALING VIA NFKB
HALLMARK P53 PATHWAY
HALLMARK INTERFERON GAMMA RESPONSE
HALLMARK INTERFERON ALPHA RESPONSE
HALLMARK INFLAMMATORY RESPONSE
HALLMARK IL6 JAK STAT3 SIGNALING
HALLMARK IL2 STAT5 SIGNALING
HALLMARK APOPTOSIS
B
% of Up (+) and Down (–) Regulated Pathway Genes
5.0
2.5
0.0
−2.5
−5.0

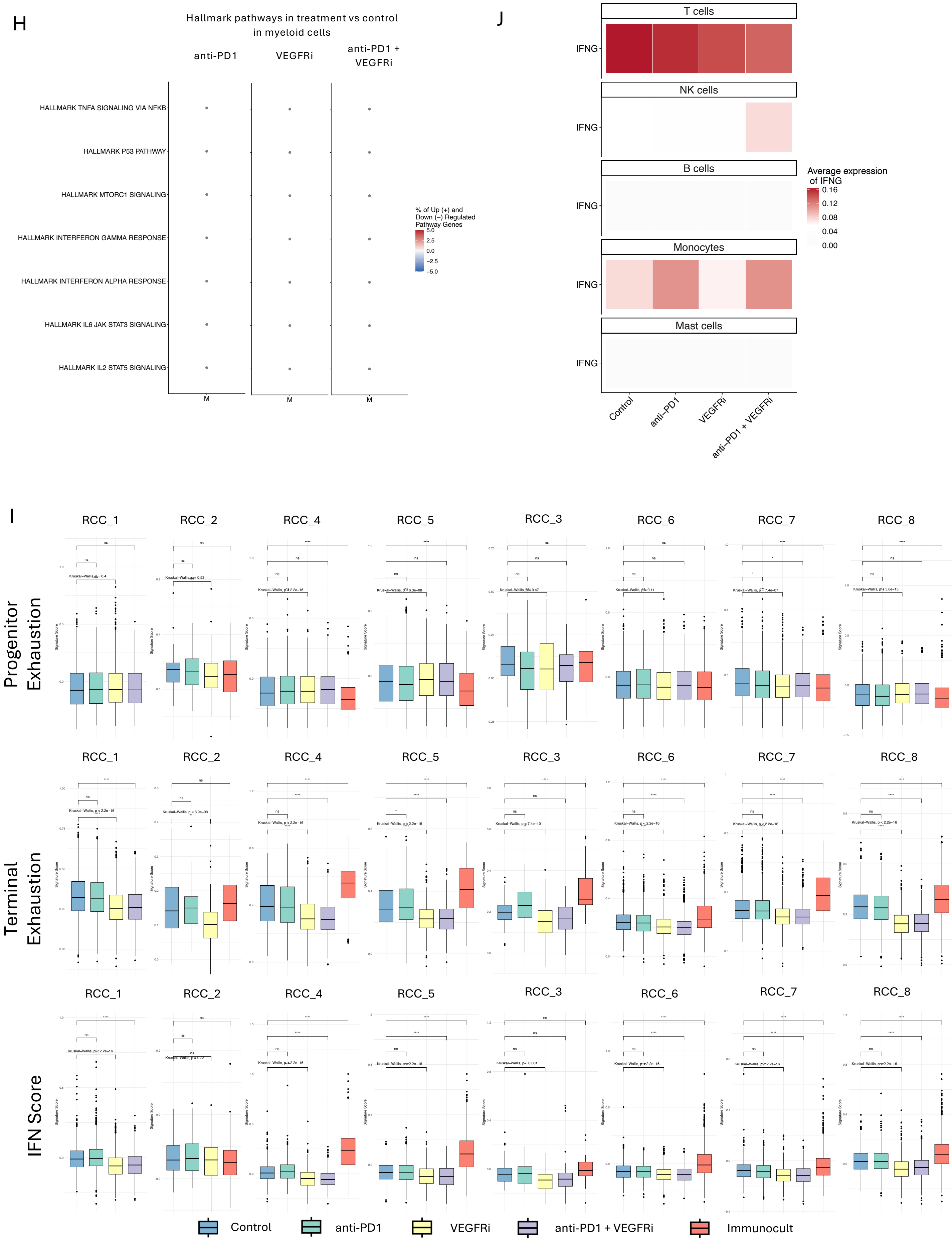
H
Hallmark pathways in treatment vs control
in myeloid cells
anti-PD1
VEGFRi
anti-PD1 +
VEGFRi
HALLMARK TNFA SIGNALING VIA NFKB
HALLMARK P53 PATHWAY
HALLMARK MTORC1 SIGNALING
HALLMARK INTERFERON GAMMA RESPONSE
HALLMARK INTERFERON ALPHA RESPONSE
HALLMARK IL6 JAK STAT3 SIGNALING
HALLMARK IL2 STAT5 SIGNALING
M
M
M
% of Up (+) and
Down (–) Regulated
Pathway Genes
5.0
2.5
0.0
−2.5
−5.0
J
T cells
NK cells
B cells
Monocytes
Mast cells
IFNG
Average expression
of IFNG
0.16
0.12
0.08
0.04
0.00
Control
anti–PD1
VEGFRi
anti–PD1 + VEGFRi
I
RCC_1
RCC_2
RCC_4
RCC_5
RCC_3
RCC_6
RCC_7
RCC_8
Progenitor
Exhaustion
Terminal
Exhaustion
IFN Score
Signature Score
Control
anti-PD1
VEGFRi
anti-PD1 + VEGFRi
Immunocult

Supplementary Fig. S5. Anti-PD1 (Pembrolizumab), VEGFR inhibitor (Axitinib) and their combination treatment responses in the RCC patient-derived *ex vivo* model (related to Fig. 5). (A) Heatmap displaying log2 converted difference (treatment - control) in cytokine concentration following treatments for each individual (RCC_9 - RCC_17). Patients' treatment responses are clustered using Wards clustering. (B, C) CyTOF analysis of treatment responses in CD8+ (B) and CD4+ (C) T cells for selected markers. Shown is percentage of marker positive cells in control and treatment condition. Each dot represents an individual (RCC_9 - RCC_17). Treatment induced changes in percentage of marker positive cells lacked statistical significance. (D) Dot plot (i) and feature plot (ii) of VEGF receptors and ligands. Showing a sparse expression of VEGF receptors in monocyte population and expression of some of the VEGF ligands in mast cell population. (E-H) Dot plot of immunology-related hallmark pathways, comparing treatment and control conditions in CD8+ and CD4+ T cells (E), NK cells (F), B cells (G) and myeloid cells (H). Genes were identified using the FindMarkers method and included if the p-value was < 0.05. Circle size represents the LogP value, while color indicates the percentage of genes in our dataset that are upregulated (+) (in red) or downregulated (-) (in blue). (I) Signature score distributions for terminally exhausted, progenitor exhausted and IFN signatures comparing control and treatment condition in CD8+ T cells shown for each patient (RCC_1 - RCC_8). Significance of differential signature enrichment (p value) between subtypes was determined by Kruskal-Walis test. (J) Heatmap displaying the average expression of *IFNG* in control and treatment conditions, highlighting that the average expression of *IFNG* in among "broad" cell subsets.
