# Supplementary material for: Functionally Characterizing the Renal Cell Carcinoma Tumor-Immune Microenvironment via Patient-Derived Ex Vivo Models
Source: Cancer Res Commun. 2026 Feb 26;6(2):402–20. doi: 10.1158/2767-9764.CRC-25-0447 (PMC13138221; doi:10.1158/2767-9764.CRC-25-0447)
Supplement: Supplementary Fig. S6 — Analysis of T cell clonotypes (related to Fig. 6) [file crc-25-0447_supplementary_fig.s6_suppsf6.pdf]

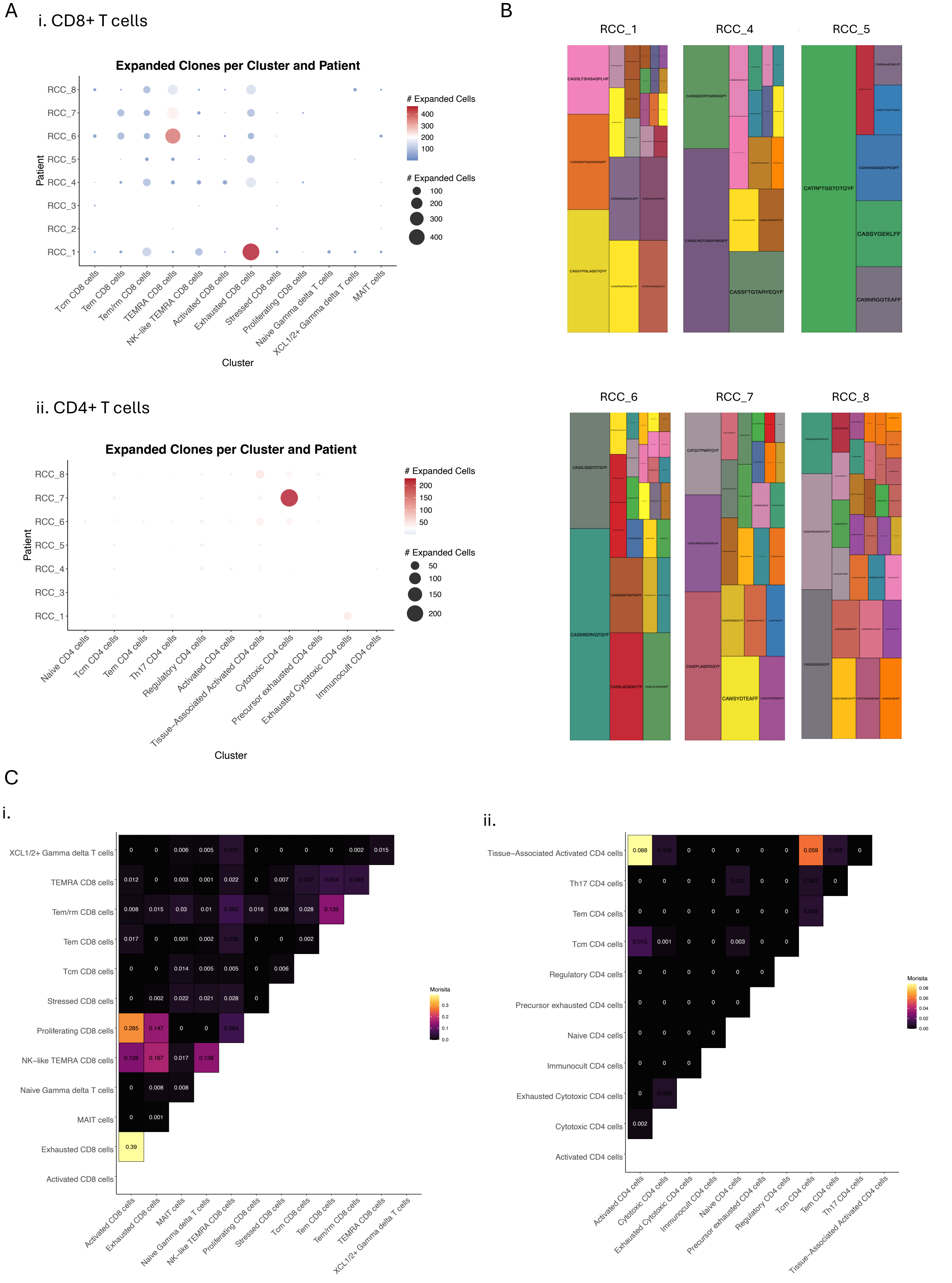

Supplementary Fig. S6

Supplementary Fig. S6. Analysis of T cell clonotypes (related to Fig. 6). (A) Dot plot showing the abundance of expanded clones among RCC patients (RCC_1 - RCC_8) in CD8+ (i) and CD4+ (ii) T cells, per cluster. (B) Tree plot showing the largest clonotypes per patient and their relative abundance. Clonotypes are marked by their variable TCR B (TRB) gene CDR3 sequence. (C) Heatmap showing similarities between different clusters in CD8+ (i) and CD4+ (ii) T cells.
