# Supplementary material for: Functionally Characterizing the Renal Cell Carcinoma Tumor-Immune Microenvironment via Patient-Derived Ex Vivo Models
Source: Cancer Res Commun. 2026 Feb 26;6(2):402–20. doi: 10.1158/2767-9764.CRC-25-0447 (PMC13138221; doi:10.1158/2767-9764.CRC-25-0447)
Supplement: Supplementary Fig.S7 — Cell-cell communication in RCC TME (related to Fig 7.) [file crc-25-0447_supplementary_fig.s7_suppsf7.pdf]

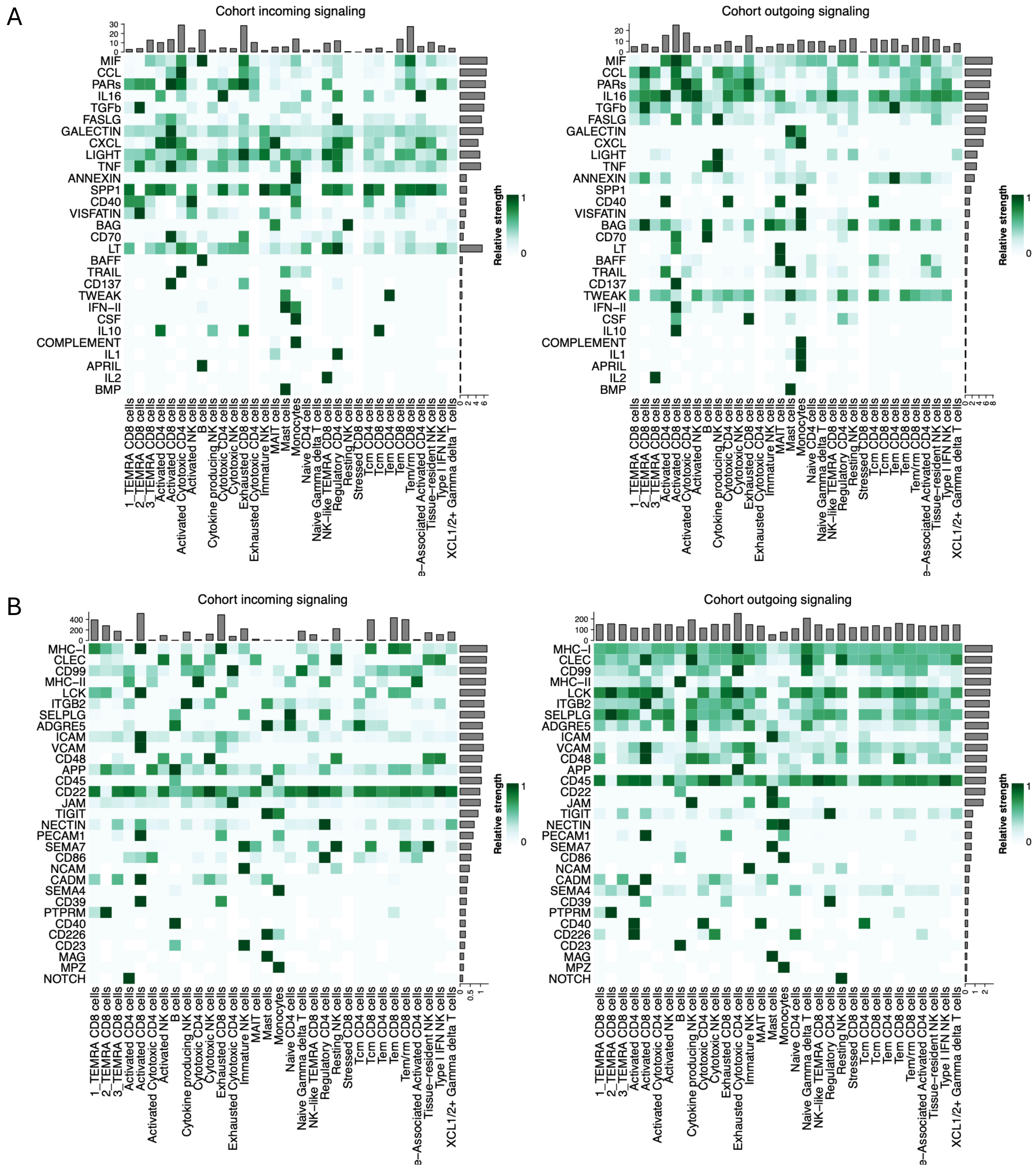

Supplementary Fig. S7

C

Ligand expression 'source'

Receptor expression 'target'

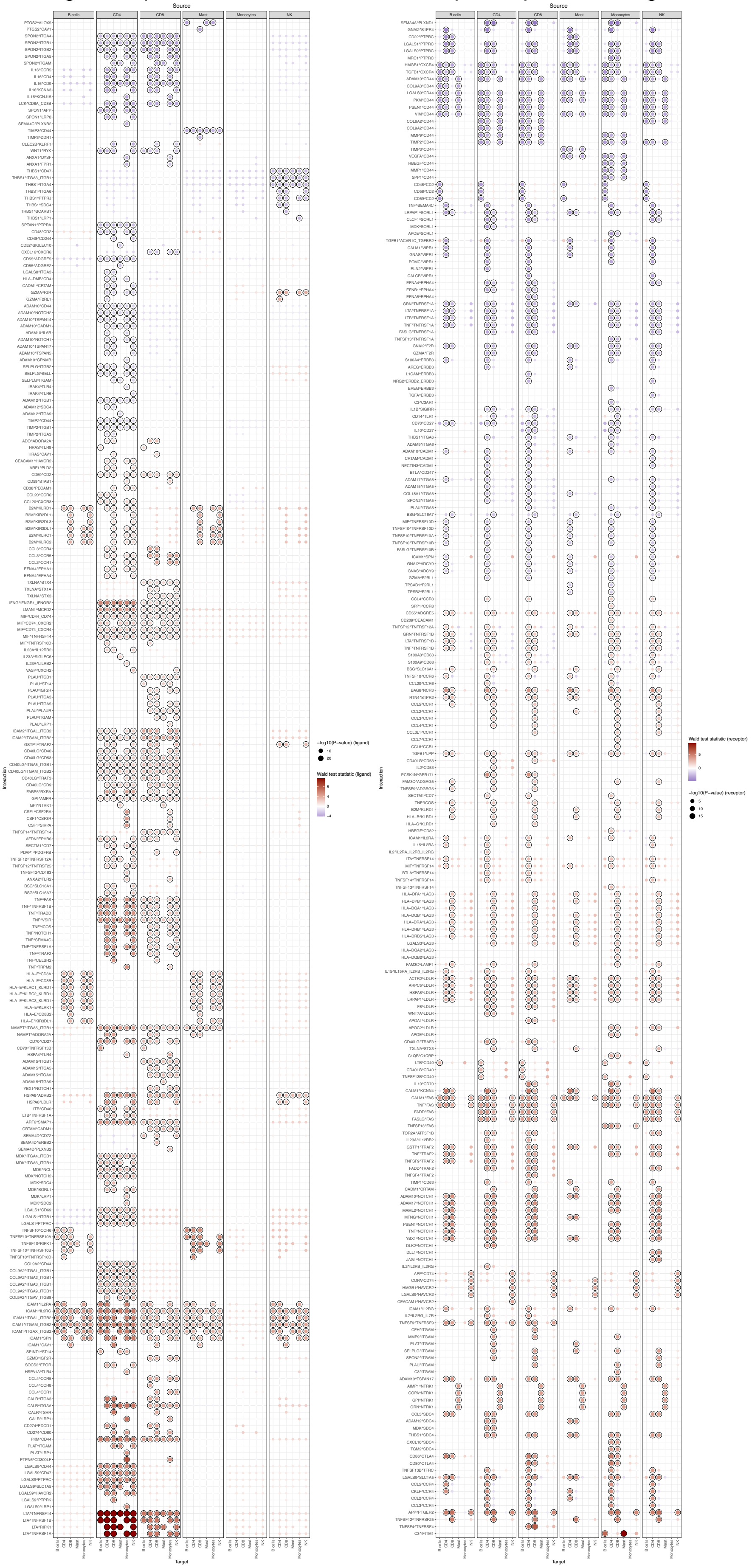

D

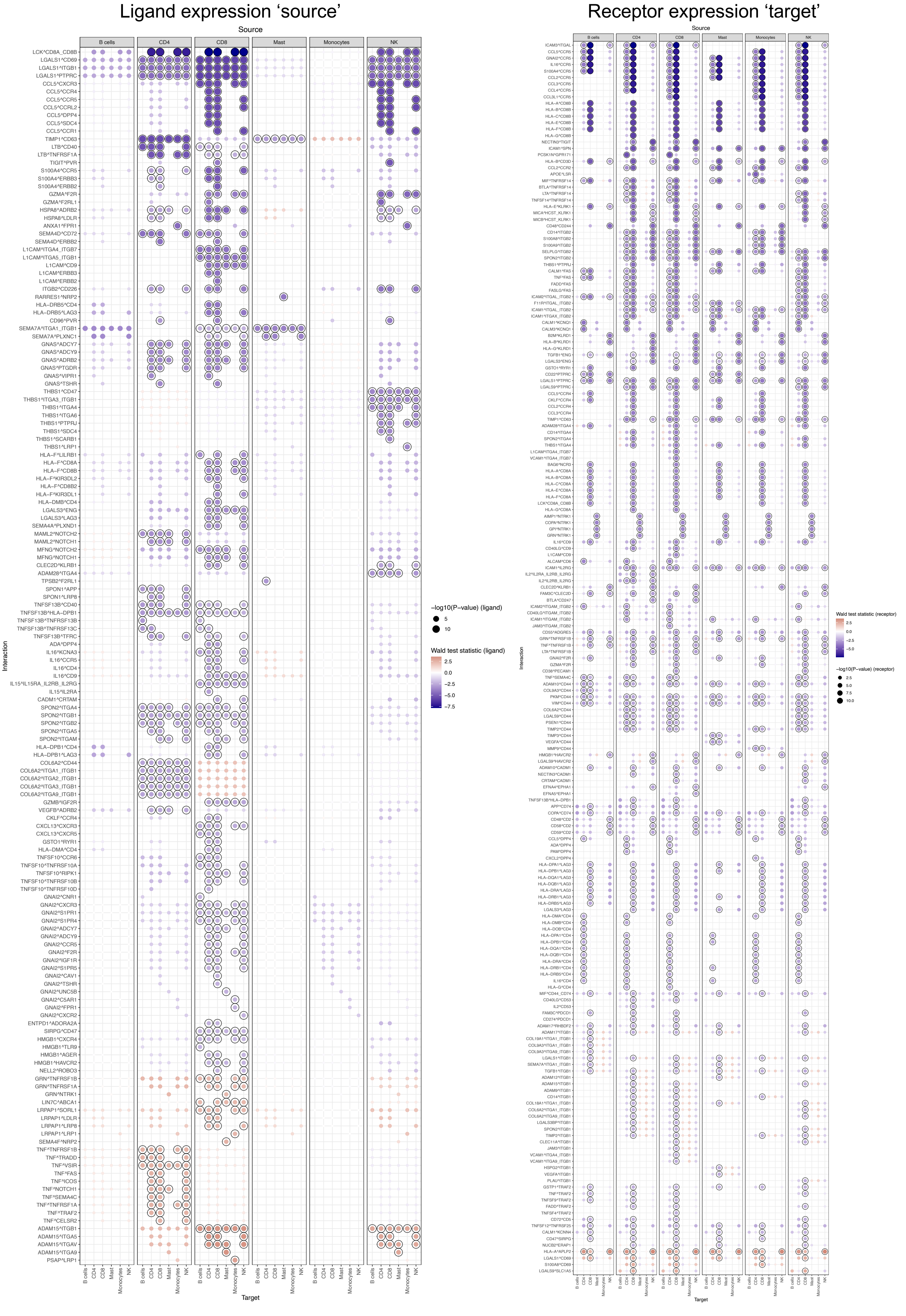

Ligand expression 'source'
Receptor expression 'target'
Source
B cells
CD4
CD8
Mast
Monocytes
NK
Target
Interaction
-log10(P-value) (ligand)
5
10
Wald test statistic (ligand)
2.5
0.0
-2.5
-5.0
-7.5
Wald test statistic (receptor)
2.5
0.0
-2.5
-5.0
-log10(P-value) (receptor)
2.5
5.0
7.5
10.0

E

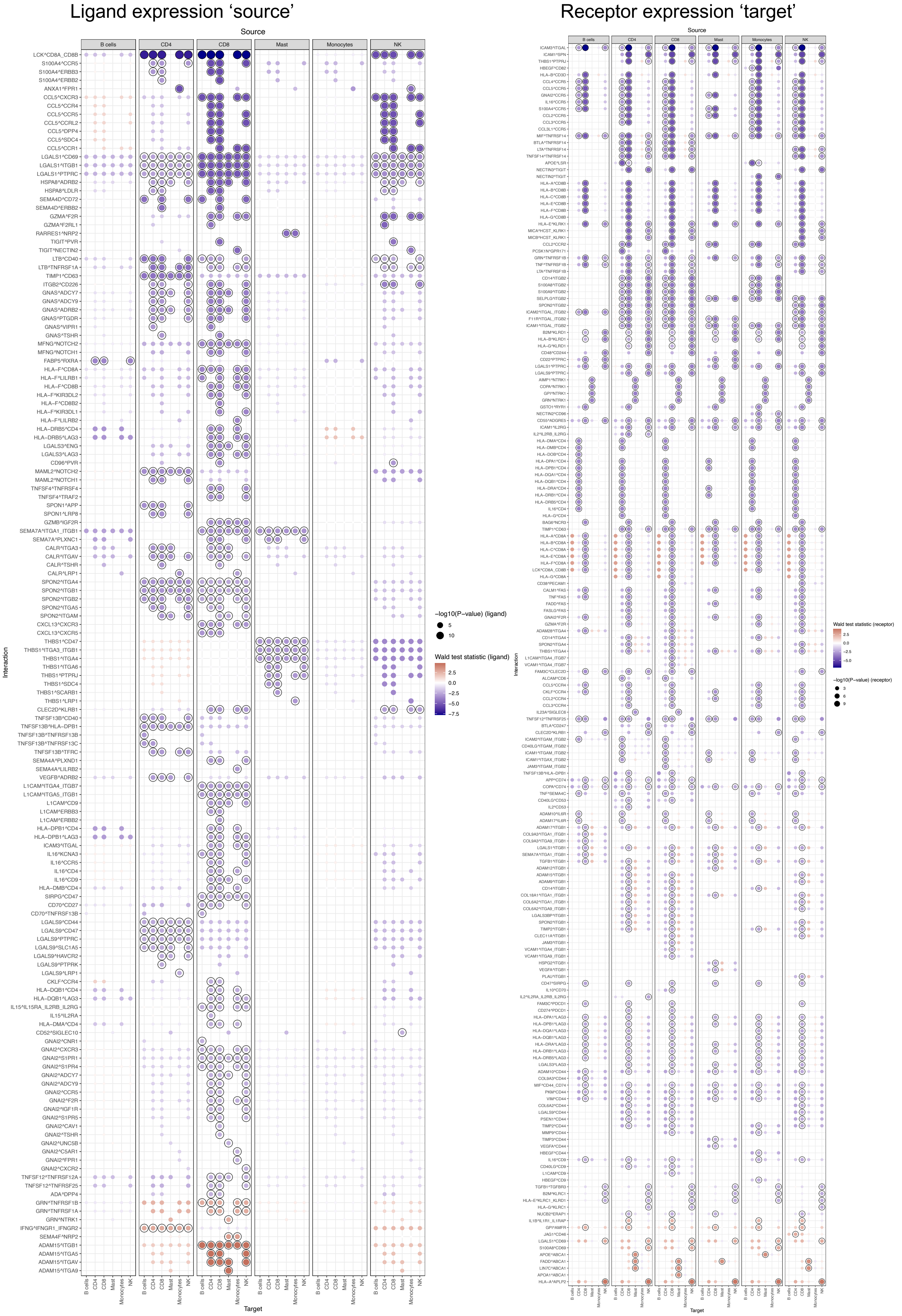

Ligand expression ‘source’
Receptor expression ‘target’
Source
B cells
CD4
CD8
Mast
Monocytes
NK
Interaction
Target
LCK^CD8A_CD8B
S100A4^CCR5
S100A4^ERBB3
S100A4^ERBB2
ANXA1^FPR1
CCL5^CXCR3
CCL5^CCR4
CCL5^CCR5
CCL5^CCRL2
CCL5^DPP4
CCL5^SDC4
CCL5^CCR1
LGALS1^CD69
LGALS1^ITGB1
LGALS1^PTPRC
HSPA8^ADRB2
HSPA8^LDLR
SEMA4D^CD72
SEMA4D^ERBB2
GZMA^F2R
GZMA^F2RL1
RARRES1^NRP2
TIGIT^PVR
TIGIT^NECTIN2
LTB^CD40
LTB^TNFRSF1A
TIMP1^CD63
ITGB2^CD226
GNAS^ADCY7
GNAS^ADCY9
GNAS^ADRB2
GNAS^PTGDR
GNAS^VIPR1
GNAS^TSHR
MFNG^NOTCH2
MFNG^NOTCH1
FABP5^RXRA
HLA−F^CD8A
HLA−F^LILRB1
HLA−F^CD8B
HLA−F^KIR3DL2
HLA−F^CD8B2
HLA−F^KIR3DL1
HLA−F^LILRB2
HLA−DRB5^CD4
HLA−DRB5^LAG3
LGALS3^ENG
LGALS3^LAG3
CD96^PVR
MAML2^NOTCH2
MAML2^NOTCH1
TNFSF4^TNFRSF4
TNFSF4^TRAF2
SPON1^APP
SPON1^LRP8
GZMB^IGF2R
SEMA7A^ITGA1_ITGB1
SEMA7A^PLXNC1
CALR^ITGA3
CALR^ITGAV
CALR^TSHR
CALR^LRP1
SPON2^ITGA4
SPON2^ITGB1
SPON2^ITGB2
SPON2^ITGA5
SPON2^ITGAM
CXCL13^CXCR3
CXCL13^CXCR5
THBS1^CD47
THBS1^ITGA3_ITGB1
THBS1^ITGA4
THBS1^ITGA6
THBS1^PTPRJ
THBS1^SDC4
THBS1^SCARB1
THBS1^LRP1
CLEC2D^KLRB1
TNFSF13B^CD40
TNFSF13B^HLA−DPB1
TNFSF13B^TNFRSF13B
TNFSF13B^TNFRSF13C
TNFSF13B^TFRC
SEMA4A^PLXND1
SEMA4A^LILRB2
VEGFB^ADRB2
L1CAM^ITGA4_ITGB7
L1CAM^ITGA5_ITGB1
L1CAM^CD9
L1CAM^ERBB3
L1CAM^ERBB2
HLA−DPB1^CD4
HLA−DPB1^LAG3
ICAM3^ITGAL
IL16^KCNA3
IL16^CCR5
IL16^CD4
IL16^CD9
HLA−DMB^CD4
SIRPG^CD47
CD70^CD27
CD70^TNFRSF13B
LGALS9^CD44
LGALS9^CD47
LGALS9^PTPRC
LGALS9^SLC1A5
LGALS9^HAVCR2
LGALS9^PTPRK
LGALS9^LRP1
CKLF^CCR4
HLA−DQB1^CD4
HLA−DQB1^LAG3
IL15^IL15RA_IL2RB_IL2RG
IL15^IL2RA
HLA−DMA^CD4
CD52^SIGLEC10
GNAI2^CNR1
GNAI2^CXCR3
GNAI2^S1PR1
GNAI2^S1PR4
GNAI2^ADCY7
GNAI2^ADCY9
GNAI2^CCR5
GNAI2^F2R
GNAI2^IGF1R
GNAI2^S1PR5
GNAI2^CAV1
GNAI2^TSHR
GNAI2^UNC5B
GNAI2^C5AR1
GNAI2^FPR1
GNAI2^CXCR2
TNFSF12^TNFRSF12A
TNFSF12^TNFRSF25
ADA^DPP4
GRN^TNFRSF1B
GRN^TNFRSF1A
GRN^NTRK1
IFNG^IFNGR1_IFNGR2
SEMA4F^NRP2
ADAM15^ITGB1
ADAM15^ITGA5
ADAM15^ITGAV
ADAM15^ITGA9
−log10(P−value) (ligand)
5
10
Wald test statistic (ligand)
2.5
0.0
−2.5
−5.0
−7.5
Wald test statistic (receptor)
2.5
0.0
−2.5
−5.0
−log10(P−value) (receptor)
3
6
9

Supplementary Fig. S7. Cell-cell communication in RCC TME (related to Fig. 7). (A, B) Heatmap showing aggregated cell-cell communication network among patients cell clusters and signalling pathways for secreted signalling (A) and cell-cell contact (B), showing signal for cell types as 'senders' (outgoing signalling) and 'receivers' (incoming signalling). Interaction probabilities were computed separately for each individual using CellChat and were further used to compute network centrality scores to infer intercellular communication at the level of signalling pathways. Color bar represents relative signalling strength of a signalling pathway across cell clusters, values are row-scaled. The top bar shows total signalling strength of a cell cluster by summarizing all cell clusters displayed in the heatmap. The right bar shows normalized signalling strength of a pathway by summarizing all cell clusters and taking natural logarithm and negative reciprocal value displayed in the heatmap. (C-E) Treatment-induced change in ligand and receptor expression in 'source' and 'target' cells, respectively, analysed using LIANA+. The results of the LIANA+ pseudobulk analysis comparing each treatment to the control condition were visualized as a dot plot. Dot plot shows upregulated ligands and receptors in red and downregulated in blue (as measured using Wald test statistic), dot size reflects -log10P value. Treatments are anti-CD3/CD28/CD2 (C), VEGFRi (D) and anti-PD1 + VEGFRi (E).
